# Unraveling the role of mobile genetic elements in antibiotic resistance transmission and defense strategies in bacteria

**DOI:** 10.3389/fsysb.2025.1557413

**Published:** 2025-08-08

**Authors:** Ranjith Kumavath, Puja Gupta, Eswar Rao Tatta, Mahima S. Mohan, Simi Asma Salim, Siddhardha Busi

**Affiliations:** ^1^ Department of Biotechnology, School of Life Sciences, Pondicherry University, Puducherry, India; ^2^ Department of Biotechnology, School of Biosciences, RIMT University, Sirhind, Punjab, India; ^3^ Department of Microbiology, School of Life Sciences, Pondicherry University, Puducherry, India

**Keywords:** antibiotic resistance, mobile genetic elements, horizontal gene transfer, bioinformatics, prokaryotic defense

## Abstract

Irrational antibiotic use contributes to the development of antibiotic resistance in bacteria, which is a major cause of healthcare-associated infections globally. Molecular research has shown that multiple resistance frequently develops from the uptake of pre-existing resistance genes, which are subsequently intensified under selective pressures. Resistant genes spread and are acquired through mobile genetic elements which are essential for facilitating horizontal gene transfer. MGEs have been identified as carriers of genetic material and are a significant player in evolutionary processes. These include insertion sequences, transposons, integrative and conjugative elements, plasmids, and genomic islands, all of which can transfer between and within DNA molecules. With an emphasis on pathogenic bacteria, this review highlights the salient features of the MGEs that contribute to the development and spread of antibiotic resistance. MGEs carry non-essential genes, including AMR and virulence genes, which can enhance the adaptability and fitness of their bacterial hosts. These elements employ evolutionary strategies to facilitate their replication and dissemination, thus enabling survival without positive selection for the harboring of beneficial genes.

## 1 Introduction

Antibiotics are often called “wonder drugs” because they are incredibly effective in fighting infections. The use of antibiotics has transformed infection treatment strategies and continues to save millions of lives. However, microorganisms like bacteria and viruses are becoming resistant to drugs due to their increased use and misuse. Due to their antimicrobial resistance (AMR), these microorganisms can survive and keep spreading even in the presence of treatments crafted to eliminate them (Endale et al., 2023). Antibiotic-resistant bacteria (ARB) pose serious threats to public health because of their swift emergence and global transmission, which makes treating infectious diseases more difficult and increases the possibility of serious repercussions. The World Health Organization (WHO) has identified AMR as a vital concern for world health (Chinemerem Nwobodo et al., 2022; Willemsen et al., 2022). The primary mechanisms by which bacteria resist antimicrobials are by limiting their uptake, modifying their target, inactivating them, or through active efflux. Gram-negative bacteria generally use all of these mechanisms for drug resistance, whereas for Gram-positive bacteria, limiting antimicrobials’ entry into their cells is less frequent, and they also lack some drug efflux systems. Drug inactivation by β-lactam ring modification (with the help of β-lactamases) is a common resistance mechanism that bacteria use against β-lactam antibiotics. Genes for these enzymes may be innately found on the bacterial chromosome or acquired through mobile genetic elements (MGEs) such as plasmids. Plasmids carry a wide variety of *bla* genes (β-lactamase genes). Drug-target-site modification by enzymes coded by *erm* genes is another common drug resistance mechanism. Over 30 different *erm* genes have been identified, many of which are located in MGEs. This wide distribution accounts for their presence in various bacterial genera, including both aerobic and anaerobic Gram-positive and Gram-negative bacteria. In case of *Staphylococcus aureus*, the most significant *erm* genes are *ermA*, which is primarily found in transposons of methicillin-resistant *S. aureus* (MRSA), and *erm*(C), which is located in plasmids of methicillin-susceptible *S. aureus*. In contrast, *erm*(B) has been reported more frequently in enterococci and pneumococci and can be found in plasmids and both conjugative and non-conjugative transposons, such as Tn*917* and Tn*551*. The genes for specific efflux pumps, such as the SMR (small multidrug resistance) pump family, can also be found on plasmids and transposable elements other than in bacterial chromosomes (Munita and Arias, 2016; Reygaert, 2018). Transmission of these resistance genes located in MGEs, called “horizontal gene transfer” (HGT), is considered the primary mechanism behind the emergence of drug resistance and transmission among pathogenic bacteria, promoting the co-selection of resistance traits (Von Wintersdorff et al., 2016). It enables the rapid sharing of resistance traits across bacterial populations, driving the increase of multidrug-resistant strains. HGT is as a crucial evolutionary force, promoting the acquisition of beneficial traits like antibiotic resistance, virulence, and metabolic adaptability in prokaryotes, thereby enhancing their survival (Emamalipour et al., 2020; Liu G. et al., 2022).

The interaction between HGT and MGEs, such as transposons, integrons, plasmids, and genomic islands, allows bacteria to acquire, exchange, and spread resistance genes across diverse environments and species, thus contributing to the persistence and proliferation of resistant strains (Haudiquet et al., 2022). When one resistance gene is present on a plasmid, it can lead to the retention of other linked resistance genes, even without selective pressure for those specific genes. This co-selection can result in the advent of multidrug-resistant (MDR) bacteria, creating significant treatment and infection control challenges (Orlek et al., 2023). Understanding the origins of MGE is inherently complex, and this complexity is intensified because these elements frequently form combination patterns of various genes and sub-elements, each possessing a distinct evolutionary history. The mosaic structure of these molecules enables a wide range of interactions with other genetic components, driving exchanges that significantly enhance genetic diversity. Furthermore, they do not belong to any specific cell or lineage; instead, they maintain independent evolutionary trajectories that diverge from traditional phylogenetic trees (Stokes and Gillings, 2011). This review summarizes the role and salient features of the MGEs that contribute to antibiotic resistance development and spread and emphasizes pathogenic bacteria. Following the discussion of various MGEs contributing to AMR, we provide an overview of how MGEs interact with cell envelopes. Additionally, we summarize resistance by chromosomal mutation and horizontal gene transfer. The defensive strategies against MGEs by host bacteria are then discussed in detail. We conclude the review by introducing various bioinformatics tools for MGE and antibiotic resistance genes (ARGs) detection and monitoring.

## 2 Mobile genetic elements conferring antimicrobial resistance

**FIGURE 1:**
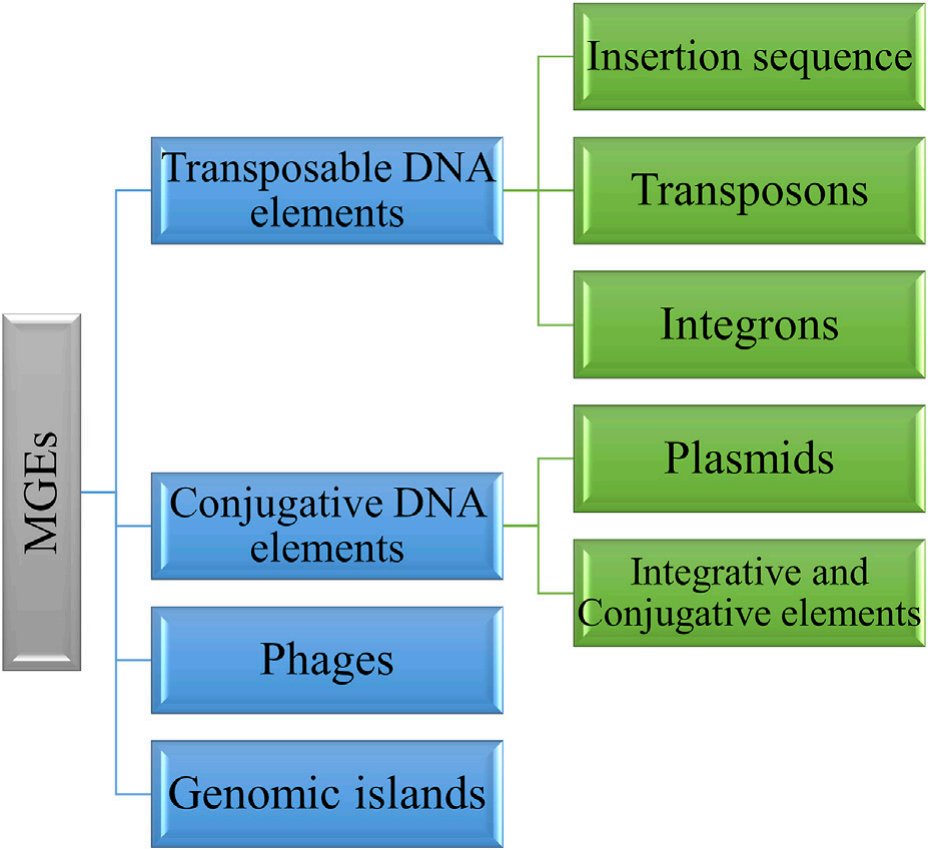
Various types of MGEs involved in antimicrobial resistance mechanisms.

MGEs exhibit diverse structure and functions. They consist of transposable DNA elements such as insertion sequence (IS), transposons and integrons, conjugative DNA elements including plasmids and integrative and conjugative elements, and phages (Tokuda and Shintani, 2024) (Figure 1). These elements promote bacterial evolution and facilitate DNA transfer between bacterial cells. However, MGEs are now identified as vectors of antimicrobial resistance genes through HGT (Ghaly and Gillings, 2022). Some MGEs can be inserted into the bacterial genome and promote chromosome-encoded resistance. The insertion can be reversible or irreversible based on the element. The irreversible insertion could lead to a loss of horizontal movement, whereas in reversible insertion, horizontal transmission is possible and remains an independent replicon. The insertion of these elements needs an integrase enzyme; either it is present within the MGEs, such as plasmids, genomic islands, or ICEs, or MGEs hijack the host chromosome for the integrase. The integrated chromosome can revert and excise from the chromosome, and the horizontal movement of MGEs depends upon the donor cell’s proximity to the recipient cells (Brown-Jaque et al., 2015).

### 2.1 Transposable DNA elements

Transposable elements (TEs) are DNA sequences that can translocate from one place to another within the DNA or to other places. The transposase enzyme is involved in the transposition mechanism. They are abundant in nature and are desirable for biological diversification. Bacterial TEs include insertion sequence (IS), transposons, and integrons. IS is the simplest and most abundant transposable element, whereas other TEs, such as transposons, vary from IS and generally hold additional genes for antibiotic resistance. They can transmit antibiotic-resistance genes within bacterial species. The insertion of IS elements causes the inactivation of genes through direct integration (Babakhani and Oloomi, 2018; Fan et al., 2019).

#### 2.1.1 Insertion sequence

**FIGURE 2:**
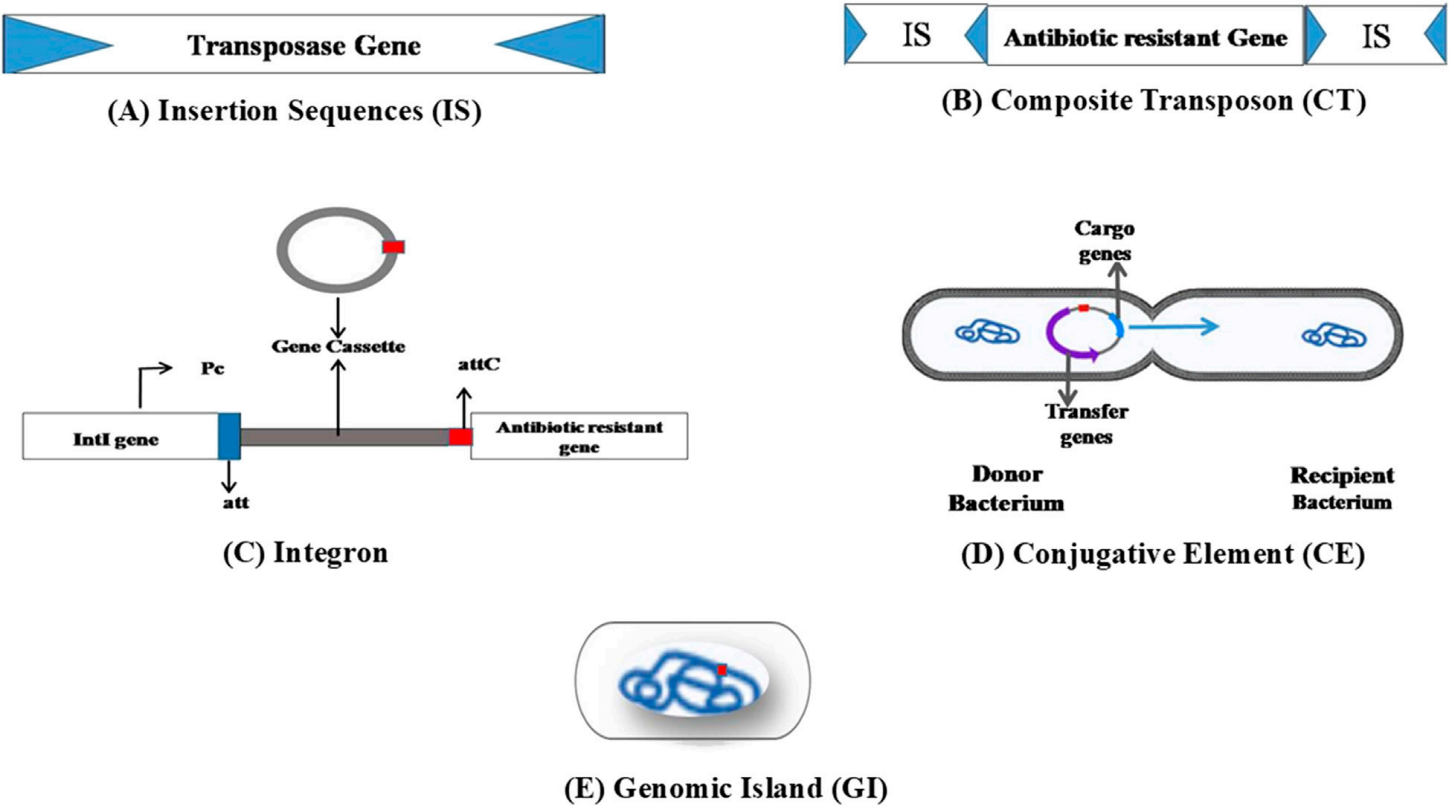
Basic structure of various MGEs involved in the spread of ARGs. **(A)** Insertion sequence composed of transposase, which is flanked by short terminal inverted repeat sequences at the ends. **(B)** Composite transposons consisting of ARGs flanked by IS at both ends. **(C)** Integrons composed of the intI gene, which codes for integrase, the attI site, responsible for primary recombination, and a promoter (Pc) region. Integrase enzyme recognizes the attI site and favors the integration of gene cassettes. **(D)** Conjugative elements are MGEs where the cargo genes are excised from the donor chromosome and enter the recipient cell through conjugation. **(E)** Genomic islands harbor MGEs flanked by repeated sequences able to excise spontaneously and transfer into the host cell.

Insertion sequence (IS) represents the simplest MGE widely distributed across all domains of life. They can relocate within a genome or by horizontal gene transfer through plasmids and phages. The size of the IS is less than 3 kb and occurs in large copy numbers in the genome. IS elements can undergo transposition by themselves, consist of short terminal inverted repeats (IR) at both ends, and have one or two open reading frame (ORF) genes that encode for transposition (Figure 2A). Around 29 IS families containing >4500 IS have been identified (Vandecraen et al., 2017). The *orf* region encodes for the IS-related transposase (Tpase), catalyzes the nucleic acid cleavage, and promotes IS mobility. Upon insertion, they often create short flanking direct repeated duplication (DR) of the target. ISfinder (https://www-is.biotoul.fr/) is a centralized database for IS elements that provides essential information related to IS nomenclature and classification. IS elements were previously represented as numbers; later, it was changed to names where the first letter is the genus name, then the first two letters of the species, followed by a number (IS*Bce1* for *Bacillus cereus*) (Siguier et al., 2015).

IS elements are classified into families based on the nature of the transposase and the catalyzes process, which includes DDE, DEDD, HUH, and Ser transposases. DDE enzymes consist of a conserved amino acid triad, such as two Asp and one Glu, and undergo a transesterification reaction using the hydroxyl group as a nucleophile. This family is abundant and widely mentioned in public databases. The catalytic domain in DDE families occurs immediately after the DNA-binding domains. The DDE family contains a transposase gene (*tnp*) flanked by terminal inverted repeats at both ends. During transposition, the Tnp protein binds at the IR and creates direct repeats on insertion, known as “target site duplications” (TSD). However, IS does not aim at sequence-specific motifs (Siguier et al., 2006; Partridge et al., 2018). DEDD transposase is related to Holliday junction resolvase, RuvC. They share a catalytic site similar to that of the DDE enzyme and are limited to only one IS family: IS*110*. They lack the terminal IR of the DDE IS, but one subgroup, IS*1111*, has sub-terminal IR; upon insertion, they do not produce target DRs. They possess a DNA-binding domain at the downstream part of the catalytic domain. HUH enzyme comprises the HUH motif, where “U” denotes the hydrophobic residue, and the Y motif contains one or two tyrosine residues. These Tpase nicks join the ssDNA using the Y motif to create a 5′ covalent bond with the substrate. Rep or replication proteins are HUH enzymes that facilitate rolling circle replication in plasmids, viruses, and phages; relaxases catalyze the conjugation and plasmid replication. HUH Tpase is commonly studied in IS*91* and IS*200*-IS*605* families (Siguier et al., 2006; Chandler et al., 2013). The IS*6* family belongs to the bacteria and archaea, which have been reported for the rearrangement and transmission of multiple antibiotic resistance. IS*26* exists in both plasmid and chromosomal components of enterobacterial clinical isolates, whereas IS*257* was found in the chromosomal and plasmid DNA of Gram-positive bacteria; both have received great interest due to their clinical impacts (Varani et al., 2021).

#### 2.1.2 Transposons

Composite transposons are ARG carriers flanked by insertion elements (Figure 2B). Transmission of ARGs by composite Tns among pathogenic bacteria makes treating infectious diseases more challenging. Tns such as Tn*903*, Tn*9*, Tn*10*, and Tn*5* carry ARGs in *Escherichia coli*. Tn*5* composite transposon consists of resistance genes encodes for kanamycin (*kan_R_*), bleomycin (*ble_R_*), and streptomycin (*str^R^*), which is flanked by IS*50*R (codes for transposase) and IS*50*L (promoter for *kan^R^* expression). Tn*5* with kanamycin resistance was reported in Gram-negative bacteria, including *Acinetobacter, Pseudomonas, Caulobacter,* and Methylobacterium (Babakhani and Oloomi, 2018). Tn*3*, a representative of unit transposons, carries antibiotic-resistance genes. Approximately 38-bp terminal-inverted repeats characterize it and contain the *tnpA* gene. Tn*3* contains the *tnpR* resolvase gene and resolution (*res*) site and may have passenger genes. These family members exhibit transposition immunity, where the transposition of an element into the same area or DNA leads to inhibition; however, homologous or *res* mediated recombination can happen between related elements, generating hybrids (Partridge et al., 2018). Transposable phage Mu of the *Myoviridae* family also disseminates resistance genes from one bacterium to another. It was identified in *E. coli* phage infection during the 1950s. Phage Mu possesses a transposition transposase enzyme and *attR* and *attL* sites at both ends. Unlike other TEs, phage Mu transfers resistance genes through lytic and lysogenic cycles (Taylor, 1963; Babakhani and Oloomi, 2018).

#### 2.1.3 Integrons

Integrons are crucial in spreading antibiotic resistance through horizontal gene transfer. Structurally, integrons comprise 5′ to 3′ conserved segments and the variable region. Their three major components are the *intI* gene, which codes for integrase, *attI* site (primary recombination site), and a promoter (Pc) (Bhat et al., 2023) (Figure 2C). Integrons acquire new genes via the gene cassettes located in the variable region. The number of gene cassettes differs; some integrons may not contain gene cassettes (Sabbagh et al., 2021; Bhat et al., 2023). Mostly, these gene cassettes acquired by integrons lack their promoters. The recombination site associated with cassettes, which wraps around a single open reading frame (ORF), is known as “*attC*” and previously as “59-base elements” (Gillings, 2014). The integrase enzyme catalyzes all the excision, integration, and rearrangements via site-specific recombination events. This enzyme can recognize the *attI* site (which, in most cases, is located upstream of *intI*), and the integration process favorably occurs at this site. The Pc promoters located upstream ensure the instant expression of the integrated new genes. In the excision process, the integrase mediates recombination between two adjacent *attC* sites (Fonseca and Vicente, 2022).

Different classification strategies are used to categorize integrons. The four distinct classes of integrons based on the *intI* gene sequence differences and divergence (Class 1–4) are well studied. Class 1 to 3 integrons are also called “resistance integrons” owing to the presence of genes responsible for antibiotic resistance. Due to their frequent association with MGEs such as transposons, resistance integrons are also called “mobile integrons” (Cury et al., 2016; Sabbagh et al., 2021; Bhat et al., 2023).

##### 2.1.3.1 Class 1 integrons

Class 1 integrons (the most frequently observed integrons) are identified initially in *Corynebacterium glutamicum* by Hall and Stokes. These integrons are commonly observed in clinical isolates of Gram-negative bacteria, including *E. coli*, *Klebsiella*, *Salmonella*, *Yersinia*, and *Shigella*, and have been identified with a minimum of 200 varied gene cassettes which confer resistance to the chloramphenicol, quaternary ammonium compounds, β-lactams, sulfonamides, aminoglycosides, quinolones, fosfomycin, trimethoprim, and other various antimicrobials (Sabbagh et al., 2021; Wang et al., 2023). Recently, Wang et al. (2023) studied the prevalence of Class 1 integrons in *Klebsiella* clinical isolates, with more than 55% of the isolates found to have these integrons. Together, these integrons carried more than 150 gene cassettes containing genes conferring resistance to various antibiotics, including carbapenems (*bla*_IPM-4_) and class D β-lactams (*bla*_OXA-1_ and *bla*_OXA-10_). The gene *qacEΔ1* (codes for an efflux pump) was shown to be the disinfectant resistance determinant in *Salmonella sp* (Chen et al., 2023). Meta-analysis of this integron class frequency in *E. coli* of urinary tract infected patients provided a global view of its occurrence. These integrons are highly prevalent in Asian countries compared to other countries Halaji et al. (2020). Liu M. et al. (2020) studied Class 1 integrons in different *P. aeruginosa* strains. The most frequent antibiotic-resistance gene cassettes were *aacA4*, *bla*_OXA-1_, and *bla*_OXA-101_.

Among Gram-positive bacteria, *S. aureus* was mostly studied for the occurrence of class 1 integrons. The presence of class 1 integrons in antibiotic-resistant *S. aureus* has been shown by various studies (Gumus et al., 2020; Zomorodi et al., 2024). In a survey by Kwiecień et al. (2020), almost 35% of *Trueperella pyogenes* isolates harbored integrons belonging to class 1. Aminoglycoside resistance genes (*aadA11* and *aadA9*) carrying gene cassettes were identified.

##### 2.1.3.2 Class 2 integrons

Class 2 integrons harboring the *intI2* integrase gene are significant in clinical isolates but less common than class 1 integrons. These integrons typically contain conserved gene cassettes, often linked to resistance against antibiotics like trimethoprim, streptomycin, and streptothricin. They are commonly associated with Tn*7*-like transposons and are prevalent in Gram-negative *Acinetobacter*, *Shigella,* and *Salmonella sp* (Yu et al., 2013; Sabbagh et al., 2021). Clinical strains of *Proteus mirabilis* were identified as having a high prevalence of class 2 integrons; more than 40% of the isolated strains harbored these integrons. For the first time, *sat2-aadA1* was found in class 2 integrons, and various other resistance cassettes were also identified (Lu et al., 2022).

##### 2.1.3.3 Class 3 integrons

Class 3 integrons are characterized by the presence of the *intI3* gene and were first recognized in *Serratia marcescens* in 1995. The *attI3* recombination site and Pc promoter are located (5′conserved segment) at a corresponding configuration like that found in class 1 integrons (Collis et al., 2002). Many bacteria, including *Acinetobacter spp., Alcaligenes, Citrobacter freundii, E. coli, K. pneumoniae, P. aeruginosa, Pseudomonas putida, and Salmonella spp*., have been reported to possess these integrons. In3-5 is a class 3 integron identified from *Enterobacter cloacae* hospital isolates (Yu et al., 2013). It carried a *bla*_oxa_ gene variant and *aac (6′)-Ib* variant (gene for gentamicin resistance). Other class 3 integrons, such as In3-1 and In3-2, possessing antibiotic-resistance gene cassettes were reported earlier in strains isolated from patients with urinary tract infections (Barraud et al., 2013).

##### 2.1.3.4 Class 4 integrons

Initially, class 4 integrons were identified in *Vibrio cholerae*. These integrons, also called “super integrons,” are a distinctive type of integron characterized by the presence of a large number of gene cassettes. It has been reported in human, animal, and even plant pathogens (*Vibrionaceae, Shewanella, Xanthomonas,* and *Pseudomonas*), but the harboring gene cassettes have major roles other than antibiotic resistance. These integrons were found to possess gene cassettes conferring resistance to chloramphenicol and fosfomycin (Rowe-Magnus and Mazel, 2002; Akrami et al., 2019).

### 2.2 Conjugative DNA elements

#### 2.2.1 Integrative and conjugative elements

Integrative and conjugative elements (ICEs) are a large class of independently transmissible MGEs with drug-resistance genes. After excision from the chromosome, they form a circular intermediate which can be uptaken by another bacterium through conjugation and then integrated and replicated with the host chromosome (Figure 2D). Several types of ICEs are reported in both Gram-positive and -negative bacteria. SXT/R391 represents the most prominent ICE family and is abundant in Gram-negative bacteria. SXT is an important ICE involved in HGT and the rearrangement of resistance genes identified in *V. cholerae*. It was initially described in the *V. cholerae* O139 MO10 strain’s chromosome from India (SXT^MO10^) in 1992, ranging approximately 100 kb. ICEs contain antibiotic-resistant genes such as *floR*, *strA&B*, *sul2*, and *dfrA18* which encode for chloramphenicol, streptomycin, sulfamethoxazole, and trimethoprim, respectively; moreover, they can translocate both chromosomal and plasmid DNA from one strain to the other. Other than *Vibrio* sp., SXT^MO10^ was also reported in γ-proteobacteria. R391 was initially identified in the clinical isolate of *Providencia rettgeri* 1967 from South Africa. Later investigation proved that it belongs to SXT through genetic and functional analyses. It resisted antibiotics, kanamycin, and heavy metals like mercury (Waldor and Mekalanos, 1994; Ceccarelli et al., 2006; Li et al., 2016). Conserved genes control SXT/R391 ICE transmission through conjugation near the *attR* attachment site; the *setR* gene codes for a lambda cI-related transcriptional repressor that hinders *setC* and *setD* gene expression. Factors such as UV light and ciprofloxacin trigger the proteolysis of the *setR* gene, followed by its inactivation. Consequently, SetC and SetD proteins form a heterocomplex and activate SXT/R391 gene expression, which is necessary for gene transmission through conjugation (Poulin-Laprade et al., 2015). Vancomycin-resistant enterococci contain van gene clusters responsible for the evolution of resistant strains through HGT. *vanA* and *vanB* clusters are ubiquitous and are part of MGEs; the *vanB* cluster comprises three alleles (*vanB1-3*), where *vanB2* is the predominant integrated fragment of the ICEs (Janice et al., 2024).

#### 2.2.2 Plasmids

These self-replicating, extrachromosomal DNA are known to possess genes that confer resistance to various antimicrobials such as aminoglycosides, β-lactams, tetracyclines, chloramphenicol, trimethoprim, sulfonamides, quinolones, and macrolides. Plasmids obtain other MGEs like IS, transposons, and integrons which help mobilize and transfer resistance genes, toxin genes, and degrading enzymes, and which promote the HGT of resistance elements between different bacterial species, genera, and kingdoms. This inter/intra-species/genera/kingdom transfer depends on the host range (narrow or broad), conjugative properties, and conjugation efficiency (Carattoli, 2013). Plasmid size varies. Recently, the MRSA isolates from Malaysia were identified with a higher occurrence of small plasmids (<5 kb). These plasmids harbored the *ermC* gene that confers resistance to streptogramin B (MLS_B_), macrolides, and lincosamides (Al-Trad et al., 2023). Larger (>30 kb) conjugative multi-resistance plasmid families pSK41 and pGO1 found in clinical staphylococcal strains are linked to the development of resistance in *S. aureus* against aminoglycosides, β-lactams, and vancomycin (Liu et al., 2013).

In Enterobacteriaceae, the Inc1 plasmid family accounts for resistance gene transfer (especially for extended-spectrum beta-lactamase) (Carattoli et al., 2021). Rozwandowicz et al. (2018) gives a detailed discussion of various plasmid groups (IncF, IncI, IncA/C, IncL, IncN, and IncH) that carry different ARGs in the Enterobacteriaceae family. In *Pseudomonas*, mega plasmids such as pBT2436 and pBT2101 were found to harbor a wide range of ARGs, including VEB, *bla*_OXA-10_, and CARB-3, which confer resistance to β-lactams, *sul1* for sulfonamides, ANT, APH and aad for aminoglycosides, *tet* genes for tetracyclines, *ermE* for macrolides, and *floR* for phenols. The efflux pump gene *mexCD-oprJ* was also identified in pBT2436 (Cazares et al., 2020).

### 2.3 Bacteriophages

Bacteriophages, or simply “phages,” are viruses that can specifically infect bacteria through two distinct life cycles. By the lysogenic cycle, phages integrate their viral genome into the host’s DNA, enabling replication alongside the bacteria. Meanwhile, in the lytic cycle, they multiply within the host cell and subsequently release new phage particles. HGT mechanism-transduction is mediated by these independently replicating viruses. Bacteriophages are found to be associated with the transmission of ARGs through transduction (Balcazar, 2014).

Wang et al. (2018) studied the presence of different clinically significant ARG types in both bacteria and phages isolated from pig feces. They found that phage DNA contained almost 35% of the target ARG types, and certain ARGs (*sul1*, *bla*_TEM_, and *ermB*) were found in all the bacteriophage DNA samples. *ermB* was the most prevalent ARG observed in the phage genome. Bacteriophage samples from different environments subjected to ARG analysis and intI gene presence revealed the *tetA*, *intI1*, *intI2*, *intI3*, *tetW*, and *bla*_OXA-2_ and *bla*_TEM_ genes. Higher occurrence was found for *bla*_TEM_ (Anand et al., 2016). Phage DNA fractions from wastewater treatment plants and hospital wastewater were found to be detected with ARGs such as *bla*_TEM_, *bla*_SHV_, *bla*_CTX-M_, *bla*_CMY_, *mecA*, *vanA*, and *mcr-1*. Among these, *bla* were more frequent (Pires et al., 2023).

### 2.4 Genomic islands

Genomic islands are large DNA portions (generally >10 kb in size) with various gene clusters and are characterized by specific G + C content. They are located on the bacterial genome itself (Figure 2E). Mostly, these elements are flanked by repeated sequences and harbor MGEs, including plasmids and IS. Studies showed that genomic islands are frequently found to be associated with tRNA-encoding genes. Some of these elements have the ability to spontaneously excise on their own from the chromosome and transfer to appropriate recipients. These elements thus contribute to HGT along with other MGEs (Dobrindt et al., 2004; Li and Wang, 2021). *Salmonella* genomic island 1 (SGI1) is a well-studied 43-kb genomic island that possesses more than 40 coding sequences. These islands harbor genes that confer resistance to ampicillin, chloramphenicol, florfenicol, streptomycin, spectinomycin, sulfonamides, and tetracycline (Doublet et al., 2003).

Antibiotic resistance transmission through genomic islands in various other organisms has also been studied. For instance, ARGs including *mdtG, tetM, dfrG, lnuG*, and *fexA* were observed in genomic islands of multidrug-resistant *Enterococci* (Li and Wang, 2021). Genome analysis of one epidemic strain of *P. aeruginosa* revealed many novel genomic islands that harbored various antibiotic-resistance genes (Jani et al., 2016). In *S. aureus,* the cassette chromosome *mec* (SCC*mec*) specifically harbors ARGs (Ito et al., 2003). Genomic islands that accumulate and spread ARGs in the escape pathogen *A. baumannii* are AbaR-type genomic islands (AbaRs) (Bi et al., 2019). Likewise, *Trueperella pyogenes* isolates contain genomic islands with resistance genes for tetracycline and macrolides. These islands also include type IV secretion systems and transposons supporting HGT (Dong et al., 2020). Qin et al. (2012) identified genomic islands that possess different aminoglycoside resistance genes in *Campylobacter coli*. In an enteroaggregative hemorrhagic *E. coli* O104 strain, the genomic island 3 GI3 carried *bla*_TEM-1_, *sul2*, *strAB*, *tet(A)*A, and *dfrA7* genes respectively encoding resistance to ampicillin, sulfamethoxazole, streptomycin, tetracycline, and trimethoprim (Chowdhury et al., 2015).

## 3 Mobile genetic elements and cell envelope interactions

The interaction of a component of the recipient cell envelope with the tip of a conjugative pilus or the tail of a phage initiates horizontal gene transfer. Phages attach to cells using receptor-binding proteins (RBPs) which facilitate phage adhesion and stabilization on the cell surface prior to DNA injection (Bertozzi Silva et al., 2016). The range of hosts and bacterial susceptibility is determined by these RBPs, which are very specific to bacterial receptors. On the other hand, conjugation relies on some cell envelope receptors (Pérez-Mendoza and de la Cruz, 2009). Due to these distinctions, phages often have smaller host ranges.

MGE entry into the cell is restricted by structures on the cell envelope, such as the bacterial capsule. Capsules made of polysaccharide chains serve as the first contact point for phages. They can be thick and protect bacteria from various agents, including phages, by concealing phage receptors (Whitfield et al., 2020; Labrie et al., 2010). Nevertheless, certain phages can use the capsule for adsorption by accessing the outer membrane through the capsule depolymerase in their RBPs (Knecht et al., 2020). This specificity can limit these phages to infecting only bacteria with certain capsules, leading to serotype-specific phage infection and more frequent gene flow within similar serotypes (Haudiquet et al., 2021). Capsulated bacteria can be more vulnerable to specific phages. Although non-capsulated bacteria might be resistant to certain phages due to lack of a capsule, they can still be highly susceptible to conjugative transfer of genes. Therefore, non-capsulated bacteria may be more prone to receiving genetic material through conjugation but are protected against some phages (Haudiquet et al., 2021). Therefore, capsule composition influences phage and conjugation-driven gene flows. ARGs and virulence factors are carried by MGEs (plasmids and transposons) that are acquired by *Klebsiella pneumoniae* (Kpn), a gut commensal and significant public health concern (Navon-Venezia et al., 2017; Yang et al., 2019). This transfer is more frequent in hospital strains that are widespread and resistant to several medications, as well as hypervirulent strains that cause infections in the community. Kpn is particularly useful for studying the connection between HGT and the cell envelope because of the capsule biosynthetic pathway, a Wzy-dependent pathway similar to *E. coli* group 1 capsule synthesis. Capsule polymerase cluster (*cps*) genes span from 5′ *galF* to the 3′ *ugd* gene (Follador et al., 2016). Consequently, depending on its interaction with phages, the capsule may promote or hinder HGT. Intense phage predation can drive capsule swapping or inactivation, altering phage susceptibility (Haudiquet et al., 2021).

Other cell envelope components, like the O-antigen of lipopolysaccharide (LPS), play roles in MGE interactions. This acylated glycolipid is a prominent component of the outer membrane of Gram-negative bacteria. Phages often target LPS, which varies greatly within and across species. Phage resistance-related transitions between smooth and rough LPS types impact pathogenicity. Thus, variations in the composition of an envelope could modify gene flow networks and impact the HGT of envelope constituents (Whitfield et al., 2020; Bertozzi Silva et al., 2016).

## 4 Resistance by horizontal gene transfer and core gene mutation

Chromosomally acquired resistance, which occurs by mutation, occurs randomly and spontaneously but is rarely transferred. The canonical mechanisms of mutational resistance include the inactivation of the drug target site, blocking drug transport into the cell, modification, inactivation of the drug itself, and bypassing the inhibited step of the metabolic pathway (National Research Council Committee to Study the Human Health Effects of Subtherapeutic Antibiotic Use in Animal Feeds, 1980; Reygaert, 2018). However, research indicates that the global antibiotic resistance crisis evidently and unquestionably relies on HGT events. Various mechanisms of the HGT account for the frequent transmission of resistance genes apart from vertical inheritance (Lerminiaux and Cameron, 2019). Genetic exchange occurs with the help of MGEs that can move within or between DNA molecules. These elements include IS, transposons, integrons, and those capable of transferring between bacterial cells, such as plasmids and ICEs. These elements facilitate horizontal genetic exchange and encourage the execution and transfer of resistance genes (Partridge et al., 2018).

In conjugation, physical interaction between cells in the same environment occurs, forming a conjugation tube through which the transfer of MGE, such as plasmids, occurs. The potential of plasmids in the frequent dissemination of ARGs by extended-spectrum β-lactamase-producing *E. coli* in wastewater has been studied. Of the 35 trans conjugants having a high transfer rate of multiple resistant genes, a 100% rate of transfer was shown by *bla*_TEM_ and *bla*_CTX-M_ genes (Li et al., 2019). Various factors promote or demote the conjugative transfer of plasmids containing ARGs; a few examples include non-antibiotic drugs that promote the spread of antibiotic resistance through intra and inter-genera conjugation (Wang et al., 2021). Dadeh Amirfard et al. (2024) recently identified key factors affecting ARG dissemination through conjugation in aquatic environments. The environmentally relevant concentration of the anticancer drug paclitaxel and its derivative docetaxel significantly enhanced the conjugative transfer of plasmids carrying ARGs (Yang et al., 2022). Low concentrations of quaternary ammonium compounds also facilitated plasmid-mediated transfer of ARG (Liu et al., 2023).

Natural competence/transformation is another HGT mechanism in which bacteria incorporate extracellular genetic information via the expression of DNA incorporation machinery (Maree et al., 2022). The direct uptake of exogenous genetic materials from the surrounding environment occurs at this juncture. There is no shortage of cell-free DNA in the environment, owing to the frequent release of DNA from dead and impaired bacteria. Stabilized by organic matter, such cell-free DNA own a large number of ARGs (Wang et al., 2020). Recently, Maree et al. (2022) observed the natural transformation of SCCmec (a large MGE that includes the *mecA* gene, which provides resistance to β-lactam) in Gram-positive *S. aureus*. As discussed in the case of conjugation, recent research has focused on the factors that accelerate the dissemination of ARG through HGT. For instance, non-antibiotic pharmaceutical triclosan at environmental concentration enhanced extracellular ARGs by transformation (Lu et al., 2020). Similarly, Wang et al. (2020) also reported the role of non-antibiotic drugs in facilitating ARG transmission through transformation. Disinfection of water with chlorine also led to the inter-genera transformation of resistance genes (Jin et al., 2020).

Antimicrobial resistance occurring through transduction represents one of the major ARG dissemination mechanisms. In transduction, the viral particles transfer bacterial genes from one cell to another. Lysogenic infection causes donor bacterial DNA to be encased in a bacteriophage capsid, which later allows for transfer to a recipient cell. Recombination of the donor DNA to the recipient genome will occur (Lerminiaux and Cameron, 2019; Trofeit et al., 2023). Studies on bacteriophages isolated from chicken meat reported high chances of kanamycin resistance transmission in *E. coli* through transduction. They showed that a quarter of isolated phages can transduce resistance to one or many tested antimicrobials (Shousha et al., 2015). Five different *Staphylococcus epidermidis* phages were found capable of high-frequency transduction of antimicrobial resistance plasmids among *S. epidermidis* strains from different clonal complexes (Fišarová et al., 2021). The transduction of chromosomal ARGs among different strains of *Acinetobacter baumannii* was reported by Wachino et al. (2019). Interestingly, the chances of intergeneric transmission of *bla*_CTX-M_, *mel*, and *tetM* were also predicted through statistical analysis of the genome sequences (Gabashvili et al., 2020).

Core metabolic gene mutations leading to antibiotic resistance have not been well studied. Lopatkin et al. (2021) evaluated the metabolic mutations that confer antibiotic resistance in *E. coli*. Phenotypic and genotypic studies by mutating the *sucA* gene (encoding 2-oxoglutarate dehydrogenase) enzyme revealed that lower basal respiration blocks antibiotic-mediated induction of citric acid cycle activity. Thus, metabolic toxicity is circumvented and lethality is minimized. Chromosomal mutations leading to the reduced affinity of drug-targeting enzymes (DNA gyrase and topoisomerase IV) and augmented expression of efflux pumps dominate the major mechanisms of fluoroquinolone resistance. Nevertheless, plasmid-mediated fluoroquinolone resistance has been reported in laboratory settings. The resistance acquired through core gene mutations mostly did not affect its normal function. For example, point mutation of the *gyrA* gene causes reduced binding affinity of norfloxacin to the enzyme–DNA complex (Hooper, 2001). Resistance to daptomycin by *Enterococcus faecalis* occurs as a result of mutations of genes coding for major proteins associated with antibiotics and antimicrobial peptide stress response by the cell envelope (LiaFSR system), and phospholipid metabolism in the cell membrane (glycerophosphoryl diester phosphodiesterase and cardiolipin synthase (*cls*)) were reported by Tran et al. (2013)). While the HGT of antibiotic resistance is well studied, mutations in various core genes leading to antibiotic resistance and their HGT are little known and should be a focus of research.

## 5 Bioinformatics: detection of MGEs and ARGs

Although sequencing technologies have become common and cheap, the diverse nature of mobile elements limits their characterization completely (Zhang et al., 2024). Various bioinformatics tools can be used to analyze data from different sequencing technologies and metagenomic data (Table 1). To track this worldwide, researchers have developed various tools and databases that help the MGE detect, identify, and track their sources in diverse environments. MGEFinder is one such tool that identifies the MGEs’ integration and their site of insertion. The input is short-read sequencing data (Durrant et al., 2020). Arredondo-Alonso et al. (2023) introduced a Python package called MGE-cluster to aid in plasmid analysis. They showcased the advantages of this platform by studying the frequency of *mcr-1* (colistin resistance gene) through analyzing the plasmid data set of *E. coli*. A divergence-based tool called SKANDIVER was recently introduced for MGE identification. Here, unlike other detection tools such as MobileElementFinder and geNomad, which are based on databases, SKANDIVER measures MGEs through genome fragmentation, divergence time, and average nucleotide identity (ANI) (Zhang et al., 2024). Plenty of annotation software, including RAST, ISfinder, Resfinder, INTEGRAL, and The Transposon Registry, are dependable annotation tools for a combination of ARGs and MGEs. BacAnt is an altogether server that permits the annotation of ARGs, transposable elements, and integrons (Hua et al., 2021).

**TABLE 1:** List of bioinformatics databases/programs and tools used in the analysis of targeted AMR sequence from NGS and WGS data.

Tools	Website	Applications	References
AMRFinderPlus	https://www.ncbi.nlm.nih.gov/pathogens/antimicrobial-resistance/AMRFinder/	Uses protein annotations and assembled nucleotide sequences to find AMR genes, point mutations associated with resistance, and other gene classes.	Feldgarden et al. (2021)
GenoScreen	https://www.genoscreen.fr/fr/	Provides molecular techniques for microbial community characterization, monitoring, and diagnosis.	Colman et al. (2025)
CARD	https://card.mcmaster.ca/	Detects resistance genes, their products, and linked phenotypes.	McArthur et al. (2013)
PGAdb	http://wgmlstdb.imst.nsysu.edu.tw/	Provides detailed information on gene annotations, functional assignments, and genome organization.	Liu et al. (2016)
ResFinder 3.0	https://bioweb.pasteur.fr/packages/pack@resfinder@3.0	Resource-finding tool for antimicrobial resistance genes.	Florensa et al. (2022)
VFDB	http://www.mgc.ac.cn/VFs/	Centralized repository of information on virulence factors.	Liu B. et al. (2022)
COGs	https://www.ncbi.nlm.nih.gov/research/cog	Provides functional annotation of proteins based on evolutionary relationships.	Galperin et al. (2021)
ISFinder	https://www-is.biotoul.fr/	List of insertion sequences (IS) isolated from bacteria and archaea.	Siguier et al. (2006)
Quick GO	https://www.ebi.ac.uk/QuickGO/annotations	Provides quick and easy access to Gene Ontology (GO) annotations for proteins.	Binns et al. (2009)
Mykrobe	https://www.mykrobe.com/	Determines which drugs an infection is resistant to by analyzing the entire genome of a bacterial sample.	Hunt et al. (2019)
KEGG	https://www.genome.jp/kegg/mapper/reconstruct.html	Graphical representations of molecular interaction and reaction networks for cellular processes and pathways.	Kanehisa et al. (2017)
GO Annotation	https://go.princeton.edu/cgi-bin/GOTermMapper	Describes functions and relationships with other genes or proteins.	Huntley et al. (2015)
BioNumerics	https://www.applied-maths.com/	Finds single-nucleotide polymorphisms (SNPs) using whole genome sequences and analyzes the resulting whole genome SNP (wgSNP) matrix using clustering.	Vranckx et al. (2017)
Integrated Genomics Viewer	https://www.sanger.ac.uk/tool/artemis/	Uses genome browser and annotation tool, to find sequence characteristics, next-generation data, analysis results, and the sequence’s six-frame translation.	Thorvaldsdóttir et al. (2013)

The TELCoMB protocol, a computational approach developed recently, identifies resistors and mobiles and determines ARG–MGE colocalizations. This approach can take up both short- and long-read sequencing data inputs. It works by data pre-processing, calculations of resistome richness and relative abundance, colocalization identification, and finally, by generating figures (Bravo et al., 2024). Other tools and databases to evaluate the ARG composition and source tracking include SARG, ARGs-OAP, ARG analyzer, DeepARG, andCARD. (Peng et al., 2021).

## 6 Defending the genome: Strategies against MGEs

**FIGURE 3:**
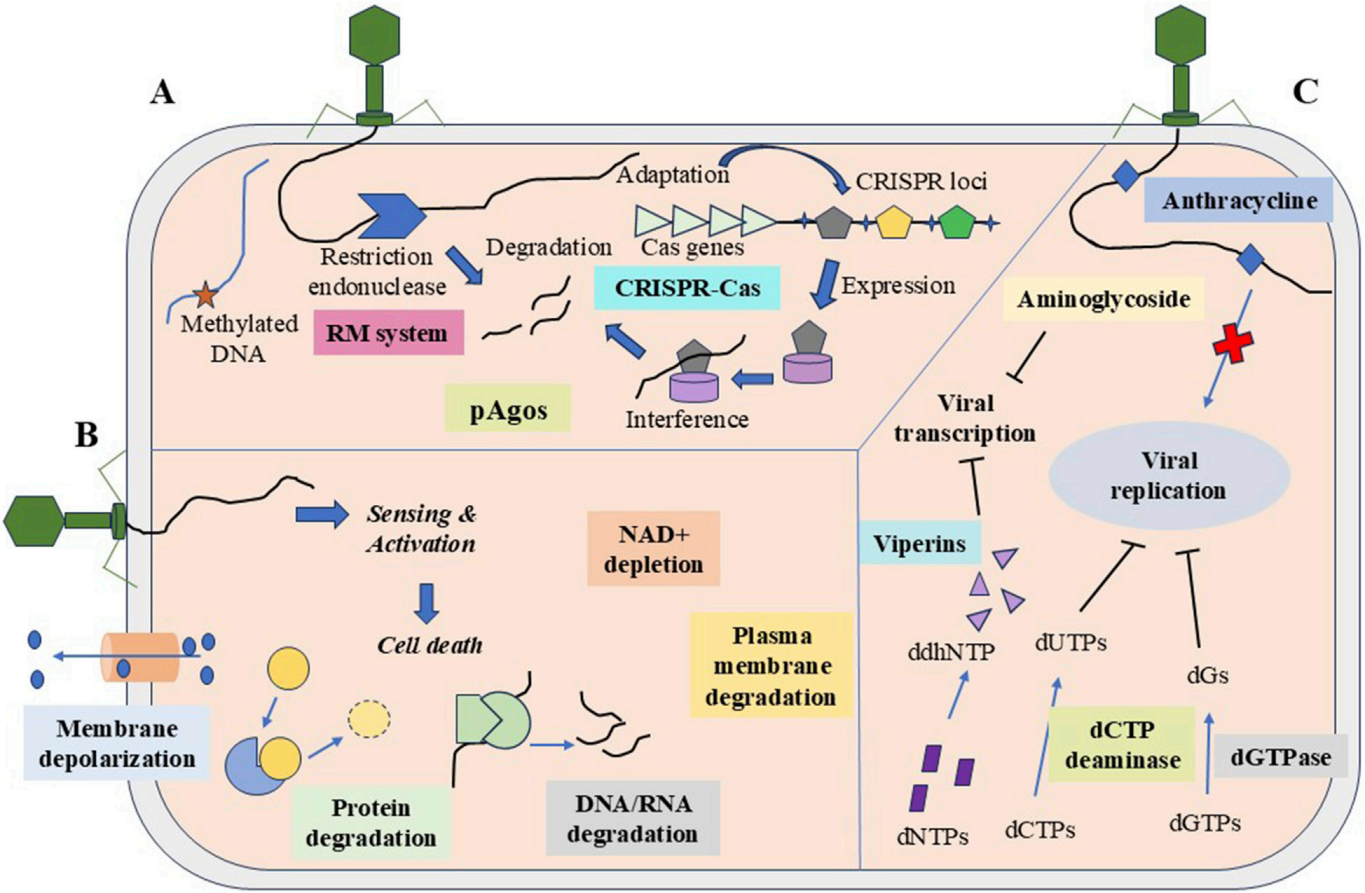
The three major strategies of the prokaryotic system against MGEs. **(A)** Degradation of nucleic acids through restriction-modification, CRISPR-Cas system, and argonaute. **(B)** Abortive infection carries out population-level protection via CBASS system, retrons, TA system, etc., and signals cell death by protein degradation, nucleic acid degradation, NAD + depletion, etc. **(C)** Inhibition of nucleic acid synthesis by chemical molecules such as anthracyclines and aminoglycosides, and the depletion of nucleotides by dCTP deaminase and dGTPase.

HGT and MGEs, such as phages/viruses and plasmids, greatly affect prokaryotic survival. To survive, prokaryotes have evolved diverse immune strategies against MGEs. The defense strategies include the degradation of genetic materials, abortive infection, and population-level protection (Mayo-Muñoz et al., 2023) (Figure 3).

### 6.1 Degradation of foreign nucleic acids

The bacterial system offers various immune strategies to defend MGEs, including nucleic acid degradation or abortive infection. The predominant defense mechanism observed in microorganisms is the degradation of invading nucleic acids (Martínez et al., 2024), which is achieved in different ways.

#### 6.1.1 Restriction modifications

The restriction modification, or RM systems, are usually regarded as the fundamental immune system of bacteria. It is the prevalent defense phenomenon observed in prokaryotes, including bacteria and archaea. These systems are well-known for cleaving foreign nucleic acids, comprising genes encoded for restriction enzymes and modification. The former, known as “restriction endonuclease,” acts as molecular scissors and cleaves the sequence lacking methyl groups; the latter is methyl transferase, involved in the methylation of the nucleic acid adenine or cytosine nucleotides. Thus, the host protects itself from endonuclease cleavage by methylation modification, while the foreign gene that lacks the group becomes vulnerable to cleavage (Liu et al., 2024; Mayo-Muñoz et al., 2023). Type I systems are hetero-oligomers with two restriction endonucleases, two methyl transferases, and one sequence-specificity region; otherwise, they have one sequence-specific unit and two methyl transferases. The type II system consists of a single homo-dimeric/tetrameric restriction endonuclease and monomeric methyl transferase, which acts independently from the other. Type III are hetero-trimers/tetramers of two genes: *res* take part in the restriction process, and *mod* functions as modification motifs. In contrast, the Type IV system cleaves modified regions of the sequence and has one or two restriction enzymes (Oliveira et al., 2014).

#### 6.1.2 CRISPR–Cas system

CRISPR and the associated prokaryotic immune system (Cas, i.e., CRISPR-associated) represent adaptive immune systems in bacteria and archaea. This system functions as defense machinery that targets extraneous DNA, such as viruses and MGEs, and independently cuts the gene sequence (Liu et al., 2024). The CRISPR–Cas loci consist of 2–100 direct, often partly palindromic repeated (25–35 bp) CRISPR arrays, parted by spacers 30–40 bp long and a set of Cas-associated genes (Koonin and Makarova, 2019).

This system includes incorporating the phage part or MGE genome into the CRISPR array as a spacer and flanking regions expressed as small CRISPR RNA (crRNA). This crRNA combines with Cas proteins, resulting in nuclease activity. The effector protein, Cas alone, cannot degrade the foreign DNA; however, in combination with crRNA, it activates the Cas, leading to the cleavage of invader DNA (Sontheimer and Davidson, 2017). This complex principally targets DNA and blocks the HGT process. Moreover, RNA was also an occasional target of the Cas–crRNA protein complex. Prokaryotes can rapidly respond to the invader genome through the reminiscence and recognition capacity of the system (Liu et al., 2024). Upon cleavage, the CRISPR–Cas system generates blunt-end fragments (Graver et al., 2024).

Based on the structure and function of the Cas protein, the CRISPR-Cas system is classified into classes I and II and is further subcategorized into types I–VI (Liu Z. et al., 2020). Type IV CRISPR-Cas systems are generally present in plasmids and sometimes in prophages (Koonin and Makarova, 2019). However, the knowledge related to this system remains unclear. Like other systems, type IV contains CRISPR arrays with spacer content, but they are devoid of Cas1-Cas2 adaptation modules. They are mostly associated with conjugative elements such as plasmids and ICEs. Recently, RNA-guided type IV CRISPR-Cas targeting was reported in *Pseudomonas oleovorans*, with a DinG helicase-dependent transcriptional repression of chromosomal targets. The CRISPR Interference system forms an R loop between crRNA and the target. DinG helicase blocks the replication fork progression by directing the R-loop. The CRISPRi system stops the targeted gene expression by blocking the binding of transcription factors or the elongation process (Benz et al., 2024).

#### 6.1.3 Argonaute

Argonaute, or Ago proteins, generally exist in three domains of life. Agos are a conserved family of nucleases that target DNA or RNA using small-guide oligonucleotides 16–21 bp long. The 5′ end of the DNA or RNA guides is hydroxylated or phosphorylated to target its substrates. Upon pairing the guides with the complementary sequence of the invader, the endonucleolytic activity of the pAgos is activated, and the phosphodiester bond of the substrate is cleaved. They do not have any known restriction on the target DNA and create staggered sticky or blunt ends during cleavage. Prokaryotic Argonautes form three phylogenetic groups: long A, long B, and short pAgos. Long Agos usually comprise amino-terminal, PAZ (PIWI-Argonaute-Zwille), L1, MID (middle domain), and PIWI (P-element induced wimpy testis) domains, which contribute to defense against foreign MGEs in prokaryotes (pAgos)—specifically DNA and RNA silencing in eukaryotes (eAgos). The long pAgos are structurally similar to eAgos. The PIWI domain is essential for nucleolytic activity. It also directs divalent metal cations via a catalytic amino acid tetrad that cuts the substrate through the nucleophilic attack, whereas inactive long pAgos often undergo mutation in the tetrad, further suppressing the endonuclease function (Zaremba et al., 2022; Graver et al., 2024). Long pAgos like CbAgo and TtAgo (from *Thermus thermophilus*) contain the catalytic domain PIWI that targets DNA using DNA guidance. Argonautes differentiate non-self- from self-DNA through many elements, including AT bases of the invader, chromatin state, gene copy number, replication frequency, and Chi sites. pAgos protect the prokaryotes from the invading DNA. However, the mechanism behind the synthesis and specificity of guide nucleotides toward the targets remains unknown (Liu et al., 2024; Graver et al., 2024; Agapov et al., 2024b). AmAgo, a member of a new group of RNA-guided pAgos from *Alteromonas macleodii*, is encoded with RNA endonuclease from the HEPN family, the Ago-associated protein Agap-HEPN. This protein splits the bond between adenine and guanine nucleotides of the RNA and produces hydroxylated 5’ -A guide RNA attached with AmAgo *in vitro* studies. *In vivo*, Agap and AmAgo acquire guide RNAs and suppress the bacteriophage attack (Agapov et al., 2024b). Recently, two defense systems, DdmABC and DdmDE, were found in *Vibrio cholerae* strains that protect the bacteria from plasmid DNAs. Using cryo-electron microscopy, researchers identified the mechanism behind the plasmid elimination by DdmDE. They reported that DdmE acts as a DNA-guided pAgos that stimulates the effector protein (DdmD) and initiates the helicase and nuclease function to cleave the invader DNA (Loeff et al., 2024).

#### 6.1.4 Gabija

The Gabija defense complex mainly comprises two components, GajA and GajB proteins, and acts as a prokaryotic virus defense system. GajA functions as a DNA endonuclease, while the role of GajB remains unclear. The Gabija defense system is broadly disseminated in bacteria and archaea and covers approximately 8.5% of the 4,360 sequenced genomes analyzed. Recent work shows that GajA requires GajB to initiate its function by sensing DNA termini synthesized by GajA to hydrolyze nucleotides. The ratio of GajA and GajB to acquire stable, functional Gabija complexes both *in vitro* and *in vivo* is 1:1. Hence, this system creates antiviral defense via nucleotide degradation and DNA nicking (Cheng et al., 2023).

### 6.2 Sequence independent strategy

Some bacterial defense systems work independently in a nucleic acid sequence to inhibit MGE transmission. The Wadjet system in bacteria cleaves the plasmid DNA and protects itself from the attack of MGEs. Approximately 6% of the sequenced bacterial genomes contain a gene Wadjet system (*jetABCD*). Proteins such as JetA, JetB, and JetC have similar sequences to that of the bacterial condensin complex MukBEF (which belongs to the SMC superfamily). The JetD protein has sequence homology to topoisomerase-primase (toprim) nucleases. The combination of SMC complex having endonucleolytic activity with Wadjet may identify and execute plasmid via DNA cleavage and loop extrusion (Deep et al., 2022). Rather than sequencing, this system discriminates the plasmids, depending on their size and circularity. Smaller closed circular plasmids stall and stimulate cleavage (Horne et al., 2023).

#### 6.2.1 Abortive infection

Upon recognition of the phage infection, the bacterial defense system induces cell death to protect other cells from the infection, before the phage matures and upholds the bacterial population (Martínez et al., 2024). This is accomplished via the exhaustion of molecules such as ATP, NAD, bacterial membrane disruption, and translation inhibition (Agapov et al., 2024a). Previously, *E. coli* widely served as a model system to study phage infections and the mechanism behind the Abi system in defense. More than 20 defense systems were described in *Lactococcus lactis* as Abi (AbiA to AbiZ) to restrict phage infection (Lopatina et al., 2020).

The first Abi system reported in *E. coli* was Rex, composed of two defense genes: *rexA* and *rexB*. This system hindered plaque formation in lambdoid and some T*7*, T*4*, and T*5* phage strains. The RexA protein sensed the presence of a protein–DNA complex during phage replication; RexA (two copies) activates one RexB protein, a membrane-bounded protein with four transmembrane helices. Following activation, RexB develops an ion channel in the inner membrane, leading to the loss of membrane potential and ATPs. This ultimately suppresses bacterial growth and halts the phage infection (Lopatina et al., 2020).

#### 6.2.2 Signaling-based defenses

Many pathways trigger the Abi system through intracellular signaling (Mayo-Muñoz et al., 2023). The cyclic oligonucleotide-based antiphase signaling system (CBASS) is a prevalent antiphase defense machinery in prokaryotes such as bacteria and archaea. More than 5,000 such operons were identified in prokaryotes, triggering an antiphase response through a secondary messenger signaling mechanism. These operons encode for two to four protein components which act as an independent system. The cGAS/DncV-like nucleotidyltransferase (CD-NTase) identifies viral replication, triggers, and catalyzes the nucleotide secondary messenger synthesis. CD-NTase binds to the CD-NTase-associated protein (Cap) and activates and induces cell death via various mechanisms. In *V. cholerae*, the second messenger molecule, cyclic dinucleotide 3′3′-c-cGAMP, consists of two purine bases synthesized by DncV (Duncan-Lowey and Kranzusch, 2022). Another Abi system involved in phage defense is PifA, found in *E. coli*, which aborts the T7 phage infection. Other systems, such as Lit and PrrC, are involved in the Abi mechanism via deactivation of host translation and inducing cell death. The defective e14 prophage of *E. coli* K12 codes for the Lit protease. The PrrC gene belongs to secondary defense and is activated once the first line of defense is distorted. PrrC audits the regular functioning of restriction enzymes by binding with type I restriction endonuclease *Eco*prrI and is activated when restriction enzymatic activity alters (Lopatina et al., 2020).

#### 6.2.3 Retrons

Retrons function as security for the immune system and help in bacterial community survival during phage infections. They are usually composed of reverse transcriptase (RT), non-coding RNA (ncRNA), and effector proteins (Liu et al., 2024). ncRNA functions as a template for RT to generate an RNA–DNA hybrid. The biological role of retrons remains unknown. Some studies suggested their role in cell specialization, pathogenesis, and starvation. However, the evidence and mechanisms behind such functions of retrons are unclear (Millman et al., 2020).

#### 6.2.4 Toxin–antitoxin system

The TA system is abundant in bacteria and archaea and is mainly composed of stable and unstable antitoxin parts. The former is a protein and the latter is an RNA or protein (Qiu et al., 2022). The functions of the TA system are plasmid maintenance, protection against phage through Abi infection, and persistence. The plasmid-encoded TA loci have been extensively studied, but those chromosomally encoded remain unexplored (Peltier et al., 2020).

#### 6.2.5 Gasdermins

Gasdermins belong to the pore-forming protein family involved in pyroptosis—host-directed cell suicide against pathogens and homeostasis maintenance in mammals. Upon detection of pathogenic determinants such as phage, LPS, and damage-associated molecular patterns (DAMPs), non-canonical caspase 11 inflammasome cleaves gasdermin D, removes the C-terminal domain, and yields a pore-forming N-terminal domain (GSDMD-NT) that oligomerizes and creates pores in the plasma membrane. It also leads to swelling and cell lysis due to a drop in osmotic pressure and water molecule entry. Studies report that cGAS- and STING-like proteins shield prokaryotes from phage infections (Liu N. et al., 2022; Zheng and Daskalov, 2023).

#### 6.2.6 Lamassu

Lamassu defends plasmids, phages, and others through the Abi mechanism, protecting the prokaryotes from infection. Several host defense systems use SMC (structural maintenance of chromosomes) like proteins, such as Lamassu, thus signifying their inherent capacity to act as DNA sensors throughout evolution. The Lamassu family encodes for three genes: *lmuA,* *lmuB,* and *lmuC*. The LmuA protein is composed of an amino-terminal Cap4 dsDNA endonuclease domain, but the function of the *lmuC* gene is unknown. In *V. cholerae*, type II lamassu called “DdmABC” shields the bacteria from plasmid replication and phage infection through Abi. A previous study proposed that LmuB recognizes phage invasion by detecting replication intermediates. Following the hydrolysis of ATP by LmuB ATPase, LmuA activates through a mechanism identical to SbcCD, the SMC-containing DNA repair system. Furthermore, detecting impaired DNA induces an associated nuclease (Mayo-Muñoz et al., 2023; Jaskólska et al., 2022; Millman et al., 2022).

#### 6.2.7 Non-catalytic pAgos

Non-catalytic pAgos involved in Abi systems, such as Long-B pAgo, short pAgo, and siAgo-like systems, lack the catalytic domains to cleave nucleic acids, unlike long-A pAgo (Olijslager et al., 2024). Long-A and -B pAgos have a similar core structure with N-terminal, L1, PAZ, L2, MID, and PIWI domains. However, the latter lacks the DEDX tetrad motif with a nuclease function (Cheng et al., 2024).

### 6.3 Inhibition of nucleic acid synthesis

#### 6.3.1 Chemical defense system

Bacteria synthesize small chemical molecules to overcome phage encounters. The major antiphage molecules that bacteria synthesize are anthracyclines, aminoglycosides, and chain terminators. Aminoglycosides have antibacterial and antiphage activities (Hardy et al., 2023). Viperin, a protein induced by interferons in animals, is known to hinder viral replication by producing chain terminator (3′-deoxy-3′,4′-didehydro (ddh)-cytidine triphosphate (ddhCTP)) for RNA polymerase. Like eukaryotic viperin, prokaryotic viperins secrete ribonucleotide, ddhCTP, that shields phage infection by halting the transcription process (Bernheim et al., 2021).

#### 6.3.2 Depletion of nucleotides

Depletion of nucleotides is another strategy to bypass phage infection. During viral replication, many deoxynucleotides are needed. Like the human antiviral factor SAMHD1, the bacterial defense system degrades dNTPs to counteract phage replication. Bacteria secrete deoxycytidine triphosphate (dCTP) deaminase, and deoxyguanosine triphosphate (dGTPase) cleaves dCTP/dGTP into deoxyuracil nucleotides and phosphate-free deoxyguanosine, respectively, leading to the starvation of nucleotides essential for the phage replication (Tal et al., 2022).

## 7 Future perspectives

The advent of sequence-based quantification methods has helped overcome time-consuming and laborious conventional methods for antibiotic resistance detection. It is now inevitable that the continuous monitoring of ARGs in the environment, animals, and humans is necessary to achieve the One Health approach objectives. A better understanding of MGEs, associated ARGs, and their transmission efficiencies is necessary to consider them as therapeutic targets, using them as biomarkers of antimicrobial resistance and other factors. However, research related to these possibilities is early and is working on overcoming its limitations. For instance, various MGE-associated genes were studied for their efficiency as biomarkers of antibiotic resistance. In addition, the *int1* gene, along with various other ARGs and crAssphage, was evaluated for its efficiency as antibiotic resistance detection and monitoring of wastewater and its downstream water samples (Teixeira et al., 2023). Biomarker studies focusing on the clinical environment are limited. Therefore, future studies focusing on this perspective will aid in early detection and monitoring and, thereby, the effective management of AMR by informing hospital and health administrations in any specific locality about the most suitable antibiotics to use and those best to avoid. This strategy can improve antibiotic treatment efficacy as well as help avoid favorable selection for MGEs in the local environment (Ghaly and Gillings, 2022). Recently, crAssphage has gained attention as a biomarker for the detection of human fecal contamination or contamination of anthropogenic origin. Some studies have considered this bacteriophage as an MGE and its association with ARGs in order to detect antibiotic resistance and monitor it (Teixeira et al., 2023; Morales-Cortés et al., 2024).

Scientific discourse is ongoing regarding the consideration of MGEs as a potential therapeutic target. Since conjugation is one of the major dissemination mechanisms of plasmids and other MGEs—including conjugative transposons and ICEs—the design or discovery of different conjugation inhibitors would be instrumental in blocking HGT. The crucial enzymes involved in conjugation (relaxase, site-specific recombinases) were studied by various researchers to potentially inhibit the spread of ARG (Vrancianu et al., 2020). Through various gene editing techniques (GETs), genes harbored on MGEs can be silenced to prevent the transmission of multidrug resistance. Integrons, the major MGEs associated with AMR in Enterobacteriaceae, were targeted by researchers to silence their resistance genes using CRISPR-Cas9 (Shetty et al., 2023). Advanced research related to MGEs is promising in the detection and monitoring of ARGs across various environments, prophylactic detection, and to exploit as a therapeutic target.

## 8 Conclusion

The evolution of antimicrobial resistance is a threat to both the environment and human health. AMR can significantly diminish the effectiveness of antibiotics, putting humans at risk of these life-saving medications no longer being effective. Transmission of AMR is due to the mobility of resistance genes through horizontal gene transfer, largely dependent on the microbial communities and mode of interactions. Most ARGs are localized with MGEs, which can translocate within the bacterial genome, the same species, or different genera. The process of horizontal gene transfer related to antibiotic resistance is still not fully understood, but it profoundly impacts people’s health everywhere. Therefore, it is important to study MGEs in depth and consider them possible targets for developing new therapeutic strategies. By using next-generation sequencing technology and various bioinformatics tools and databases, we can deepen our understanding of the mechanisms behind the dissemination of resistance.

## References

[B1] Agapov A. Baker K. S. Bedekar P. Bhatia R. P. Blower T. R. Brockhurst M. A. (2024a). Multi-layered genome defences in bacteria. Curr. Opin. Microbiol. 78, 102436. 10.1016/j.mib.2024.102436 38368839

[B2] Agapov A. Panteleev V. Kropocheva E. Kanevskaya A. Esyunina D. Kulbachinskiy A. (2024b). Prokaryotic Argonaute nuclease cooperates with co-encoded RNase to acquire guide RNAs and target invader DNA. Nucleic Acids Res 52 (10), 5895–5911. 10.1093/nar/gkae345 38716875 PMC11162769

[B3] Akrami F. Rajabnia M. Pournajaf A. (2019). Resistance integrons; A mini review. Casp J intern Med. 10 (4), 370–376. 10.22088/cjim.10.4.370 PMC6856922 31814933

[B4] Al-Trad E. A. I. Chew C. H. Che Hamzah A. M. Suhaili Z. Rahman N. I. A. Ismail S. (2023). The plasmidomic landscape of clinical methicillin-resistant *Staphylococcus aureus* isolates from Malaysia. Antibiotics 12 (4), 733. 10.3390/antibiotics12040733 37107095 PMC10135026

[B5] Anand T. Bera B. C. Vaid R. K. Barua S. Riyesh T. Virmani N. (2016). Abundance of antibiotic resistance genes in environmental bacteriophages. J. Gen. Virol. 97 (12), 3458–3466. 10.1099/jgv.0.000639 27902329

[B6] Arredondo-Alonso S. Gladstone R. A. Pöntinen A. K. Gama J. A. Schürch A. C. Lanza V. F. (2023). Mge-cluster: a reference-free approach for typing bacterial plasmids. Nar. Genom. Bioinform. 5 (3), lqad066. 10.1093/nargab/lqad066 37435357 PMC10331934

[B7] Babakhani S. Oloomi M. (2018). Transposons: the agents of antibiotic resistance in bacteria. J. Basic Microbiol. 58 (11), 905–917. 10.1002/jobm.201800204 30113080

[B8] Balcazar J. L. (2014). Bacteriophages as vehicles for antibiotic resistance genes in the environment. PLoS Pathog 10 (7), e1004219. 10.1371/journal.ppat.1004219 25078987 PMC4117541

[B9] Barraud O. Casellas M. Dagot C. Ploy M. C. (2013). An antibiotic-resistant class 3 integron in an *Enterobacter cloacae* isolate from hospital effluent. Clin Microbiol Infect 19 (7), E306–E308. 10.1111/1469-0691.12186 23458448

[B10] Benz F. Camara-Wilpert S. Russel J. Wandera K. G. Čepaitė R. Ares-Arroyo M. (2024). Type IV-A3 CRISPR-Cas systems drive inter-plasmid conflicts by acquiring spacers in trans. Cell Host Microbe 32 (6), 875–886.e9. 10.1016/j.chom.2024.04.016 38754416

[B11] Bernheim A. Millman A. Ofir G. Meitav G. Avraham C. Shomar H. (2021). Prokaryotic viperins produce diverse antiviral molecules. Nature 589 (7840), 120–124. 10.1038/s41586-020-2762-2 32937646 PMC7610908

[B12] Bertozzi Silva J. Storms Z. Sauvageau D. (2016). Host receptors for bacteriophage adsorption. FEMS Microbiol. Lett. 363 (4), fnw002. 10.1093/femsle/fnw002 26755501

[B13] Bhat B. A. Mir R. A. Qadri H. Dhiman R. Almilaibary A. Alkhanani M. (2023). Integrons in the development of antimicrobial resistance: critical review and perspectives. Front. Microbiol. 14, 1231938. 10.3389/fmicb.2023.1231938 37720149 PMC10500605

[B14] Bi D. Xie R. Zheng J. Yang H. Zhu X. Ou H. Y. (2019). Large-scale identification of AbaR-type genomic islands in *Acinetobacter baumannii* reveals diverse insertion sites and clonal lineage-specific antimicrobial resistance gene profiles. Antimicrob. Agents Chemother. 63 (4), e02526. 10.1128/AAC.02526-18 30917986 PMC6437538

[B15] Binns D. Dimmer E. Huntley R. Barrell D. O'donovan C. Apweiler R. (2009). QuickGO: a web-based tool for Gene Ontology searching. Bioinformatics 25 (22), 3045–3046. 10.1093/bioinformatics/btp536 19744993 PMC2773257

[B16] Bravo J. E. Slizovskiy I. Bonin N. Oliva M. Noyes N. Boucher C. (2024). The TELCoMB protocol for high‐sensitivity detection of ARG‐MGE colocalizations in complex microbial communities. Curr. Protoc. 4 (10), e70031. 10.1002/cpz1.70031 39444361 PMC11620000

[B17] Brown-Jaque M. Calero-Cáceres W. Muniesa M. (2015). Transfer of antibiotic-resistance genes via phage-related mobile elements. Plasmid 79, 1–7. 10.1016/j.plasmid.2015.01.001 25597519

[B18] Carattoli A. (2013). Plasmids and the spread of resistance. Int. J. Med. Microbiol. 303 (6-7), 298–304. 10.1016/j.ijmm.2013.02.001 23499304

[B19] Carattoli A. Villa L. Fortini D. García-Fernández A. (2021). Contemporary IncI1 plasmids involved in the transmission and spread of antimicrobial resistance in Enterobacteriaceae. Plasmid 118, 102392. 10.1016/j.plasmid.2018.12.001 30529488

[B20] Cazares A. Moore M. P. Hall J. P. Wright L. L. Grimes M. Emond-Rhéault J. G. (2020). A megaplasmid family driving dissemination of multidrug resistance in Pseudomonas. Nat. Commun. 11 (1), 1370. 10.1038/s41467-020-15081-7 32170080 PMC7070040

[B21] Ceccarelli D. Salvia A. M. Sami J. Cappuccinelli P. Colombo M. M. (2006). New cluster of plasmid-located class 1 integrons in *Vibrio cholerae* O1 and a dfrA15 cassette-containing integron in Vibrio parahaemolyticus isolated in Angola. Antimicrob. Agents Chemother 50 (7), 2493–2499. 10.1128/aac.01310-05 16801431 PMC1489794

[B22] Chandler M. De La Cruz F. Dyda F. Hickman A. B. Moncalian G. Ton-Hoang B. (2013). Breaking and joining single-stranded DNA: the HUH endonuclease superfamily. Nat. Rev. Microbiol 11 (8), 525–538. 10.1038/nrmicro3067 23832240 PMC6493337

[B23] Chen S. Fu J. Zhao K. Yang S. Li C. Penttinen P. (2023). Class 1 integron carrying qacEΔ1 gene confers resistance to disinfectant and antibiotics in Salmonella. Int. J. Food Microbiol. 404, 110319. 10.1016/j.ijfoodmicro.2023.110319 37473468

[B24] Cheng F. Wu A. Li Z. Xu J. Cao X. Yu H. (2024). Catalytically active prokaryotic Argonautes employ phospholipase D family proteins to strengthen immunity against different genetic invaders. mLife 3 (3), 403–416. 10.1002/mlf2.12138 39359674 PMC11442185

[B25] Cheng R. Huang F. Lu X. Yan Y. Yu B. Wang X. (2023). Prokaryotic Gabija complex senses and executes nucleotide depletion and DNA cleavage for antiviral defense. Cell Host Microbe 31 (8), 1331–1344.e5. 10.1016/j.chom.2023.06.014 37480847

[B26] Chinemerem Nwobodo D. Ugwu M. C. Oliseloke Anie C. Al‐Ouqaili M. T. Chinedu Ikem J. Victor Chigozie U. (2022). Antibiotic resistance: the challenges and some emerging strategies for tackling a global menace. J. Clin. Lab. Anal. 36 (9), e24655. 10.1002/jcla.24655 35949048 PMC9459344

[B27] Chowdhury P. R. Charles I. G. Djordjevic S. P. (2015). A role for Tn 6029 in the evolution of the complex antibiotic resistance gene loci in genomic island 3 in enteroaggregative hemorrhagic *Escherichia coli* O104: H4. PLoS One 10 (2), e0115781. 10.1371/journal.pone.0115781 25675217 PMC4326458

[B28] Collis C. M. Kim M. J. Partridge S. R. Stokes H. W. Hall R. M. (2002). Characterization of the class 3 integron and the site-specific recombination system it determines. J. Bacteriol. 184 (11), 3017–3026. 10.1128/jb.184.11.3017-3026.2002 12003943 PMC135066

[B29] Colman R. E. Seifert M. De la Rossa A. Georghiou S. B. Hoogland C. Uplekar S. (2025). Evaluating culture-free targeted next-generation sequencing for diagnosing drug-resistant tuberculosis: a multicentre clinical study of two end-to-end commercial workflows. Lancet Infect. Dis. The. 25 (3), 325–334. 10.1016/S1473-3099(24)00586-3 PMC11871994 39486428

[B30] Cury J. Jové T. Touchon M. Néron B. Rocha E. P. (2016). Identification and analysis of integrons and cassette arrays in bacterial genomes. Nucleic acids Res 44 (10), 4539–4550. 10.1093/nar/gkw319 27130947 PMC4889954

[B31] Dadeh Amirfard K. Moriyama M. Suzuki S. Sano D. (2024). Effect of environmental factors on conjugative transfer of antibiotic resistance genes in aquatic settings. J. Appl. Microbiol. 135 (6), lxae129. 10.1093/jambio/lxae129 38830804

[B32] Deep A. Gu Y. Gao Y. Q. Ego K. M. Herzik M. A. Zhou H. (2022). The SMC-family Wadjet complex protects bacteria from plasmid transformation by recognition and cleavage of closed-circular DNA. Mol. Cell. 82 (21), 4145–4159.e7. 10.1016/j.molcel.2022.09.008 36206765 PMC9637719

[B33] Dobrindt U. Hochhut B. Hentschel U. Hacker J. (2004). Genomic islands in pathogenic and environmental microorganisms. Nat. Rev. Microbiol 2 (5), 414–424. 10.1038/nrmicro884 15100694

[B34] Dong W. L. Xu Q. J. Atiah L. A. Odah K. A. Gao Y. H. Kong L. C. (2020). Genomic island type IV secretion system and transposons in genomic islands involved in antimicrobial resistance in *Trueperella pyogenes* . Vet. Microbiol. 242, 108602. 10.1016/j.vetmic.2020.108602 32122606

[B35] Doublet B. Lailler R. Meunier D. Brisabois A. Boyd D. Mulvey M. R. (2003). Variant Salmonella genomic island 1 antibiotic resistance gene cluster in *Salmonella enterica* serovar Albany. Emerg. Infect. Dis. 9 (5), 585–591. 10.3201/eid0905.020609 12737743 PMC2972765

[B36] Duncan-Lowey B. Kranzusch P. J. (2022). CBASS phage defense and evolution of antiviral nucleotide signaling. Curr. Opin. Immunol. 74, 156–163. 10.1016/j.coi.2022.01.002 35123147

[B37] Durrant M. G. Li M. M. Siranosian B. A. Montgomery S. B. Bhatt A. S. (2020). A bioinformatic analysis of integrative mobile genetic elements highlights their role in bacterial adaptation. Cell Host Microbe 27 (1), 140–153. 10.1016/j.chom.2019.10.022 31862382 PMC6952549

[B38] Emamalipour M. Seidi K. Zununi Vahed S. Jahanban-Esfahlan A. Jaymand M. Majdi H. (2020). Horizontal gene transfer: from evolutionary flexibility to disease progression. Front. Cell Dev. Biol. 8, 229. 10.3389/fcell.2020.00229 32509768 PMC7248198

[B39] Endale H. Mathewos M. Abdeta D. (2023). Potential causes of spread of antimicrobial resistance and preventive measures in one health perspective-a review. Infect. Drug Resist. 16, 7515–7545. 10.2147/IDR.S428837 38089962 PMC10715026

[B40] Fan C. Wu Y. H. Decker C. M. Rohani R. Gesell Salazar M. Ye H. (2019). Defensive function of transposable elements in bacteria. ACS Synth. Biol. 8 (9), 2141–2151. 10.1021/acssynbio.9b00218 31375026

[B41] Feldgarden M. Brover V. Gonzalez-Escalona N. Frye J. G. Haendiges J. Haft D. H. (2021). AMRFinderPlus and the Reference Gene Catalog facilitate examination of the genomic links among antimicrobial resistance, stress response, and virulence. Sci. Rep. 11 (1), 12728. 10.1038/s41598-021-91456-0 34135355 PMC8208984

[B146] Fišarová L. Botka T. Du X. Mašlaňová I. Bárdy P. Pantůček R. (2021). Staphylococcus epidermidis phages transduce antimicrobial resistance plasmids and mobilize chromosomal islands. Msphere 6 (3), 10–1128. 10.1128/mSphere.00223-21 PMC8125051 33980677

[B42] Florensa A. F. Kaas R. S. Clausen P. T. L. C. Aytan-Aktug D. Aarestrup F. M. (2022). ResFinder–an open online resource for identification of antimicrobial resistance genes in next-generation sequencing data and prediction of phenotypes from genotypes. Microb. Genom. 8 (1), 000748. 10.1099/mgen.0.000748 35072601 PMC8914360

[B43] Follador R. Heinz E. Wyres K. L. Ellington M. J. Kowarik M. Holt K. E. (2016). The diversity of *Klebsiella pneumoniae* surface polysaccharides. Microb. Genom. 2 (8), e000073. 10.1099/mgen.0.000073 28348868 PMC5320592

[B44] Fonseca É. L. Vicente A. C. (2022). Integron functionality and genome innovation: an update on the subtle and smart strategy of integrase and gene cassette expression regulation. Microorganisms 10 (2), 224. 10.3390/microorganisms10020224 35208680 PMC8876359

[B45] Gabashvili E. Osepashvili M. Koulouris S. Ujmajuridze L. Tskhitishvili Z. Kotetishvili M. (2020). Phage transduction is involved in the intergeneric spread of antibiotic resistance-associated bla ctx-m, mel, and tetm loci in natural populations of some human and animal bacterial pathogens. Curr. Microbiol. 77, 185–193. 10.1007/s00284-019-01817-2 31754824

[B46] Galperin M. Y. Wolf Y. I. Makarova K. S. Vera Alvarez R. Landsman D. Koonin E. V. (2021). COG database update: focus on microbial diversity, model organisms, and widespread pathogens. Nucleic Acids Res 49 (D1), D274–D281. 10.1093/nar/gkaa1018 33167031 PMC7778934

[B47] Ghaly T. M. Gillings M. R. (2022). New perspectives on mobile genetic elements: a paradigm shift for managing the antibiotic resistance crisis. Philos. Trans. R. Soc. B 377 (1842), 20200462. 10.1098/rstb.2020.0462 PMC8628067 34839710

[B48] Gillings M. R. (2014). Integrons: past, present, and future. Microbiol. Mol. Biol. R. 78 (2), 257–277. 10.1128/mmbr.00056-13 PMC4054258 24847022

[B49] Graver B. A. Chakravarty N. Solomon K. V. (2024). Prokaryotic Argonautes for *in vivo* biotechnology and molecular diagnostics. Trends Biotechnol 42 (1), 61–73. 10.1016/j.tibtech.2023.06.010 37451948

[B50] Gumus D. Kalayci-Yuksek F. Bayirli-Turan D. Caliskan M. Gumus A. Ozdemir S. (2020). Presence of class I and class II integrons in methicilin resistant staphylococci and their relations with antibiotic resistance: a preliminary study from Turkey. J. Health Med. Nurs. 75. 10.7176/JHMN/75-07

[B51] Halaji M. Feizi A. Mirzaei A. Sedigh Ebrahim-Saraie H. Fayyazi A. Ashraf A. (2020). The global prevalence of class 1 integron and associated antibiotic resistance in *Escherichia coli* from patients with urinary tract infections, a systematic review and meta-analysis. Microb. Drug Resist. 26 (10), 1208–1218. 10.1089/mdr.2019.0467 32282274

[B52] Hardy A. Kever L. Frunzke J. (2023). Antiphage small molecules produced by bacteria - beyond protein-mediated defenses. Trends Microbiol 31 (1), 92–106. 10.1016/j.tim.2022.08.001 36038409

[B53] Haudiquet M. Buffet A. Rendueles O. Rocha E. P. (2021). Interplay between the cell envelope and mobile genetic elements shapes gene flow in populations of the nosocomial pathogen *Klebsiella pneumoniae* . PLoS Biol 19 (7), e3001276. 10.1371/journal.pbio.3001276 34228700 PMC8259999

[B54] Haudiquet M. de Sousa J. M. Touchon M. Rocha E. P. (2022). Selfish, promiscuous and sometimes useful: how mobile genetic elements drive horizontal gene transfer in microbial populations. Philos. Trans. R. Soc. B 377 (1861), 20210234. 10.1098/rstb.2021.0234 PMC9393566 35989606

[B55] Hooper D. C. (2001). Emerging mechanisms of fluoroquinolone resistance. Emerg Infect Dis 7 (2), 337–341. 10.3201/eid0702.010239 11294736 PMC2631735

[B56] Horne T. Orr V. T. Hall J. P. (2023). How do interactions between mobile genetic elements affect horizontal gene transfer? Curr. Opin. Microbiol. 73, 102282. 10.1016/j.mib.2023.102282 36863168

[B57] Hua X. Liang Q. Deng M. He J. Wang M. Hong W. (2021). BacAnt: a combination annotation server for bacterial DNA sequences to identify antibiotic resistance genes, integrons, and transposable elements. Front. Microbiol. 12, 649969. 10.3389/fmicb.2021.649969 34367079 PMC8343408

[B58] Hunt M. Bradley P. Lapierre S. G. Heys S. Thomsit M. Hall M. B. (2019). Antibiotic resistance prediction for *Mycobacterium tuberculosis* from genome sequence data with Mykrobe. Wellcome Open Res 4, 191. 10.12688/wellcomeopenres.15603.1 32055708 PMC7004237

[B59] Huntley R. P. Sawford T. Mutowo-Meullenet P. Shypitsyna A. Bonilla C. Martin M. J. (2015). The Goa database: gene ontology annotation updates for 2015. Nucleic Acids Res 43 (D1), D1057–D1063. 10.1093/nar/gku1113 25378336 PMC4383930

[B60] Ito T. Okuma K. Ma X. X. Yuzawa H. Hiramatsu K. (2003). Insights on antibiotic resistance of *Staphylococcus aureus* from its whole genome: genomic island SCC. Drug resist. Updat. 6 (1), 41–52. 10.1016/S1368-7646(03)00003-7 12654286

[B61] Jani M. Mathee K. Azad R. K. (2016). Identification of novel genomic islands in Liverpool epidemic strain of *Pseudomonas aeruginosa* using segmentation and clustering. Front. Microbiol. 7, 1210. 10.3389/fmicb.2016.01210 27536294 PMC4971588

[B62] Janice J. Wagner T. M. Olsen K. Hegstad J. Hegstad K. (2024). Emergence of vancomycin-resistant enterococci from vancomycin-susceptible enterococci in hospitalized patients under antimicrobial therapy. J. Glob. Antimicrob. Resist. 36, 116–122. 10.1016/j.jgar.2023.12.010 38128726

[B63] Jaskólska M. Adams D. W. Blokesch M. (2022). Two defence systems eliminate plasmids from seventh pandemic *Vibrio cholerae* . Nature 604 (7905), 323–329. 10.1038/s41586-022-04546-y 35388218 PMC7613841

[B64] Jin M. Liu L. Wang D. N. Yang D. Liu W. L. Yin J. (2020). Chlorine disinfection promotes the exchange of antibiotic resistance genes across bacterial genera by natural transformation. ISME J. 14 (7), 1847–1856. 10.1038/s41396-020-0656-9 32327733 PMC7305130

[B65] Kanehisa M. Furumichi M. Tanabe M. Sato Y. Morishima K. (2017). KEGG: new perspectives on genomes, pathways, diseases and drugs. Nucleic Acids Res 45 (D1), D353–D361. 10.1093/nar/gkw1092 27899662 PMC5210567

[B66] Knecht L. E. Veljkovic M. Fieseler L. (2020). Diversity and function of phage encoded depolymerases. Front. Microbiol. 10, 2949. 10.3389/fmicb.2019.02949 31998258 PMC6966330

[B67] Koonin E. V. Makarova K. S. (2019). Origins and evolution of CRISPR-Cas systems. Philos. Trans. R. Soc. B 374 (1772), 20180087. 10.1098/rstb.2018.0087 PMC6452270 30905284

[B68] Kwiecień E. Stefańska I. Chrobak-Chmiel D. Sałamaszyńska-Guz A. Rzewuska M. (2020). New determinants of aminoglycoside resistance and their association with the class 1 integron gene cassettes in Trueperella pyogenes. Int. J. Mol. Sci. 21 (12), 4230. 10.3390/ijms21124230 32545831 PMC7352783

[B69] Labrie S. J. Samson J. E. Moineau S. (2010). Bacteriophage resistance mechanisms. Nat. Rev. Microbiol 8 (5), 317–327. 10.1038/nrmicro2315 20348932

[B70] Lerminiaux N. A. Cameron A. D. (2019). Horizontal transfer of antibiotic resistance genes in clinical environments. Can. J. Microbiol. 65 (1), 34–44. 10.1139/cjm-2018-0275 30248271

[B71] Li Q. Chang W. Zhang H. Hu D. Wang X. (2019). The role of plasmids in the multiple antibiotic resistance transfer in ESBLs-producing *Escherichia coli* isolated from wastewater treatment plants. Front. Microbiol. 10, 633. 10.3389/fmicb.2019.00633 31001218 PMC6456708

[B72] Li W. Wang A. (2021). Genomic islands mediate environmental adaptation and the spread of antibiotic resistance in multiresistant Enterococci-evidence from genomic sequences. BMC Microbiol 21, 55–10. 10.1186/s12866-021-02114-4 33602143 PMC7893910

[B73] Li X. Du Y. Du P. Dai H. Fang Y. Li Z. (2016). SXT/R391 integrative and conjugative elements in Proteus species reveal abundant genetic diversity and multidrug resistance. Sci. Rep. 6 (1), 37372. 10.1038/srep37372 27892525 PMC5124997

[B74] Liu B. Zheng D. Zhou S. Chen L. Yang J. (2022). VFDB 2022: a general classification scheme for bacterial virulence factors. Nucleic Acids Res 50 (D1), D912–D917. 10.1093/nar/gkab1107 34850947 PMC8728188

[B75] Liu C. Goh S. G. You L. Yuan Q. Mohapatra S. Gin K. Y. H. (2023). Low concentration quaternary ammonium compounds promoted antibiotic resistance gene transfer via plasmid conjugation. Sci. Total Environ. 887, 163781. 10.1016/j.scitotenv.2023.163781 37149193 PMC10158037

[B76] Liu G. Thomsen L. E. Olsen J. E. (2022). Antimicrobial-induced horizontal transfer of antimicrobial resistance genes in bacteria: a mini-review. J. Antimicrob. Chemother. 77 (3), 556–567. 10.1093/jac/dkab450 34894259

[B77] Liu M. Ma J. Jia W. Li W. (2020). Antimicrobial resistance and molecular characterization of gene cassettes from class 1 integrons in *Pseudomonas aeruginosa* strains. Microb. Drug Resis. 26 (6), 670–676. 10.1089/mdr.2019.0406 PMC7307683 32407190

[B78] Liu M. A. Kwong S. M. Jensen S. O. Brzoska A. J. Firth N. (2013). Biology of the staphylococcal conjugative multiresistance plasmid pSK41. Plasmid 70 (1), 42–51. 10.1016/j.plasmid.2013.02.001 23415796

[B79] Liu N. Pang X. Zhang H. Ji P. (2022). The cGAS-STING pathway in bacterial infection and bacterial immunity. Front. Immunol. 12, 814709. 10.3389/fimmu.2021.814709 35095914 PMC8793285

[B80] Liu S. Liu H. Wang X. Shi L. (2024). The immune system of prokaryotes: potential applications and implications for gene editing. Biotechnol. 19 (2), 2300352. 10.1002/biot.202300352 38403433

[B81] Liu Y. Y. Chiou C. S. Chen C. C. (2016). PGAdb-builder: a web service tool for creating pan-genome allele database for molecular fine typing. Sci. Rep. 6 (1), 36213. 10.1038/srep36213 27824078 PMC5099940

[B82] Liu Z. Dong H. Cui Y. Cong L. Zhang D. (2020). Application of different types of CRISPR/Cas-based systems in bacteria. Microb. Cell Factories 19, 172–14. 10.1186/s12934-020-01431-z PMC7470686 32883277

[B83] Loeff L. Adams D. W. Chanez C. Stutzmann S. Righi L. Blokesch M. (2024). Molecular mechanism of plasmid elimination by the DdmDE defense system. Science 385, 188–194. 10.1126/science.adq0534 38870273

[B84] Lopatina A. Tal N. Sorek R. (2020). Abortive infection: bacterial suicide as an antiviral immune strategy. Annu. Rev. Virol. 7 (1), 371–384. 10.1146/annurev-virology-011620-040628 32559405

[B85] Lopatkin A. J. Bening S. C. Manson A. L. Stokes J. M. Kohanski M. A. Badran A. H. (2021). Clinically relevant mutations in core metabolic genes confer antibiotic resistance. Science 371 (6531), eaba0862. 10.1126/science.aba0862 33602825 PMC8285040

[B86] Lu J. Wang Y. Zhang S. Bond P. Yuan Z. Guo J. (2020). Triclosan at environmental concentrations can enhance the spread of extracellular antibiotic resistance genes through transformation. Sci. Total Environ. 713, 136621. 10.1016/j.scitotenv.2020.136621 32019018

[B87] Lu W. Qiu Q. Chen K. Zhao R. Li Q. Wu Q. (2022). Distribution and molecular characterization of functional class 2 integrons in clinical *Proteus mirabilis* isolates. Infect. Drug Resist. 15, 465–474. 10.2147/IDR.S347119 35210790 PMC8858760

[B88] Maree M. Thi Nguyen L. T. Ohniwa R. L. Higashide M. Msadek T. Morikawa K. (2022). Natural transformation allows transfer of SCC mec-mediated methicillin resistance in *Staphylococcus aureus* biofilms. Nat. Commun 13 (1), 2477. 10.1038/s41467-022-29877-2 35513365 PMC9072672

[B89] Martínez M. Rizzuto I. Molina R. (2024). Knowing our enemy in the antimicrobial resistance era: dissecting the molecular basis of bacterial defense systems. Int. J. Mol. Sci. 25 (9), 4929. 10.3390/ijms25094929 38732145 PMC11084316

[B90] Mayo-Muñoz D. Pinilla-Redondo R. Birkholz N. Fineran P. C. (2023). A host of armor: prokaryotic immune strategies against mobile genetic elements. Cell Rep 42 (7), 112672. 10.1016/j.celrep.2023.112672 37347666

[B91] McArthur A. G. Waglechner N. Nizam F. Yan A. Azad M. A. Baylay A. J. (2013). The comprehensive antibiotic resistance database. Antimicrob. Agents Chemother. 57 (7), 3348–3357. 10.1128/aac.00419-13 23650175 PMC3697360

[B92] Millman A. Bernheim A. Stokar-Avihail A. Fedorenko T. Voichek M. Leavitt A. (2020). Bacterial retrons function in anti-phage defense. Cell 183 (6), 1551–1561. 10.1016/j.cell.2020.09.065 33157039

[B93] Millman A. Melamed S. Leavitt A. Doron S. Bernheim A. Hör J. (2022). An expanded arsenal of immune systems that protect bacteria from phages. Cell Host Microbe 30 (11), 1556–1569.e5. 10.1016/j.chom.2022.09.017 36302390

[B94] Morales-Cortés S. Sala-Comorera L. Gómez-Gómez C. Muniesa M. García-Aljaro C. (2024). CrAss-like phages are suitable indicators of antibiotic resistance genes found in abundance in fecally polluted samples. Environ. Pollut. 359, 124713. 10.1016/j.envpol.2024.124713 39134166

[B95] Munita J. M. Arias C. A. (2016). “Mechanisms of antibiotic resistance,” in Virulence mechanisms of bacterial pathogen*s* . Editors Kuduva I. T. Cornick N. A. Plummer P. J. Zhang Q. Nicholson T. L. Bannantine J. P. (Wiley), 481–511.

[B96] National Research Council (US) Committee to Study the Human Health Effects of Subtherapeutic Antibiotic Use in Animal Feeds (1980). The effects on human health of subtherapeutic use of antimicrobials in animal feeds. Washington: National Academies Press. 25032407

[B97] Navon-Venezia S. Kondratyeva K. Carattoli A. (2017). *Klebsiella pneumoniae*: a major worldwide source and shuttle for antibiotic resistance. FEMS Microbiol. Rev. 41 (3), 252–275. 10.1093/femsre/fux013 28521338

[B98] Olijslager L. H. Weijers D. Swarts D. C. (2024). Distribution of specific prokaryotic immune systems correlates with host optimal growth temperature. Nar. Genom. Bioinform. 6 (3), lqae105. 10.1093/nargab/lqae105 39165676 PMC11333966

[B99] Oliveira P. H. Touchon M. Rocha E. P. (2014). The interplay of restriction-modification systems with mobile genetic elements and their prokaryotic hosts. Nucleic Acids Res 42 (16), 10618–10631. 10.1093/nar/gku734 25120263 PMC4176335

[B100] Orlek A. Anjum M. F. Mather A. E. Stoesser N. Walker A. S. (2023). Factors associated with plasmid antibiotic resistance gene carriage revealed using large-scale multivariable analysis. Sci. Rep. 13 (1), 2500. 10.1038/s41598-023-29530-y 36781908 PMC9925765

[B101] Partridge S. R. Kwong S. M. Firth N. Jensen S. O. (2018). Mobile genetic elements associated with antimicrobial resistance. Clin. Microbiol. Rev. 31 (4), e00088. 10.1128/cmr.00088-17 30068738 PMC6148190

[B102] Peltier J. Hamiot A. Garneau J. R. Boudry P. Maikova A. Hajnsdorf E. (2020). Type I toxin-antitoxin systems contribute to the maintenance of mobile genetic elements in Clostridioides difficile. Commun. Biol. 3 (1), 718. 10.1038/s42003-020-01448-5 33247281 PMC7699646

[B103] Peng Z. Mao Y. Zhang N. Zhang L. Wang Z. Han M. (2021). Utilizing metagenomic data and Bioinformatic tools for elucidating antibiotic resistance genes in environment. Front. Environ. Sci. 9, 757365. 10.3389/fenvs.2021.757365

[B104] Pérez-Mendoza D. de la Cruz F. (2009). *Escherichia coli* genes affecting recipient ability in plasmid conjugation: are there any? BMC genom 10, 1–14. 10.1186/1471-2164-10-71 PMC2645431 19203375

[B105] Pires J. Santos R. Monteiro S. (2023). Antibiotic resistance genes in bacteriophages from wastewater treatment plant and hospital wastewaters. Sci. Total Environ. 892, 164708. 10.1016/j.scitotenv.2023.164708 37315610

[B106] Poulin-Laprade D. Matteau D. Jacques P. E. Rodrigue S. Burrus V. (2015). Transfer activation of SXT/R391 integrative and conjugative elements: unraveling the SetCD regulon. Nucleic Acids Res 43 (4), 2045–2056. 10.1093/nar/gkv071 25662215 PMC4344509

[B107] Qin S. Wang Y. Zhang Q. Chen X. Shen Z. Deng F. (2012). Identification of a novel genomic island conferring resistance to multiple aminoglycoside antibiotics in Campylobacter coli. Antimicrob. Agents Chemother. 56 (10), 5332–5339. 10.1128/aac.00809-12 22869568 PMC3457361

[B108] Qiu J. Zhai Y. Wei M. Zheng C. Jiao X. (2022). Toxin–antitoxin systems: classification, biological roles, and applications. Microbiol. Res. 264, 127159. 10.1016/j.micres.2022.127159 35969944

[B109] Reygaert W. C. (2018). An overview of the antimicrobial resistance mechanisms of bacteria. AIMS Microbiol 4 (3), 482–501. 10.3934/microbiol.2018.3.482 31294229 PMC6604941

[B110] Rowe-Magnus D. A. Mazel D. (2002). The role of integrons in antibiotic resistance gene capture. Int. J. Med. Microbiol. 292 (2), 115–125. 10.1078/1438-4221-00197 12195734

[B111] Rozwandowicz M. Brouwer M. S. M. Fischer J. Wagenaar J. A. Gonzalez-Zorn B. Guerra B. (2018). Plasmids carrying antimicrobial resistance genes in Enterobacteriaceae. J. Antimicrob. Chemother. 73 (5), 1121–1137. 10.1093/jac/dkx488 29370371

[B112] Sabbagh P. Rajabnia M. Maali A. Ferdosi-Shahandashti E. (2021). Integron and its role in antimicrobial resistance: a literature review on some bacterial pathogens. *Iran* . J. Basic Med. Sci. 24 (2), 136–142. 10.22038/ijbms.2020.48905.11208 PMC8061329 33953851

[B113] Shetty V. P. Akshay S. D. Rai P. Deekshit V. K. (2023). Integrons as the potential targets for combating multidrug resistance in Enterobacteriaceae using CRISPR-Cas9 technique. J. Appl Microbiol. 134 (7), lxad137. 10.1093/jambio/lxad137 37410611

[B114] Shousha A. Awaiwanont N. Sofka D. Smulders F. J. Paulsen P. Szostak M. P. (2015). Bacteriophages isolated from chicken meat and the horizontal transfer of antimicrobial resistance genes. Appl. Environ. Microbiol. 81 (14), 4600–4606. 10.1128/AEM.00872-15 25934615 PMC4551174

[B115] Siguier P. Gourbeyre E. Varani A. Ton-Hoang B. Chandler M. (2015). “Everyman's guide to bacterial insertion sequences,” in Mobile DNA III. Editors Chandler M. Gellert M. Lambowitz A. M. Rice P. A. Sandmeyer S. B. (Wiley), 555–590. 10.1128/microbiolspec.MDNA3-0030-2014 26104715

[B116] Siguier P. Pérochon J. Lestrade L. Mahillon J. Chandler M. (2006). ISfinder: the reference centre for bacterial insertion sequences. Nucleic Acids Res 34 (Suppl_1), D32–D36. 10.1093/nar/gkj014 16381877 PMC1347377

[B117] Sontheimer E. J. Davidson A. R. (2017). Inhibition of CRISPR-Cas systems by mobile genetic elements. Curr. Opin. Microbiol. 37, 120–127. 10.1016/j.mib.2017.06.003 28668720 PMC5737815

[B118] Stokes H. W. Gillings M. R. (2011). Gene flow, mobile genetic elements and the recruitment of antibiotic resistance genes into Gram-negative pathogens. FEMS Microbiol. Rev. 35 (5), 790–819. 10.1111/j.1574-6976.2011.00273.x 21517914

[B119] Tal N. Millman A. Stokar-Avihail A. Fedorenko T. Leavitt A. Melamed S. (2022). Bacteria deplete deoxynucleotides to defend against bacteriophage infection. Nat. Microbiol 7 (8), 1200–1209. 10.1038/s41564-022-01158-0 35817891

[B120] Taylor A. L. (1963). Bacteriophage-induced mutation in *Escherichia coli* . Proc Natl Acad Sci U. S. A. 50 (6), 1043–1051. 10.1073/pnas.50.6.1043 14096176 PMC221271

[B121] Teixeira A. M. Vaz-Moreira I. Calderón-Franco D. Weissbrodt D. Purkrtova S. Gajdos S. (2023). Candidate biomarkers of antibiotic resistance for the monitoring of wastewater and the downstream environment. Water Res 247, 120761. 10.1016/j.watres.2023.120761 37918195

[B122] Thorvaldsdóttir H. Robinson J. T. Mesirov J. P. (2013). Integrative Genomics Viewer (IGV): high-performance genomics data visualization and exploration. Brief. Bioinform. 14 (2), 178–192. 10.1093/bib/bbs017 22517427 PMC3603213

[B123] Tokuda M. Shintani M. (2024). Microbial evolution through horizontal gene transfer by mobile genetic elements. Microb. Biotechnol. 17 (1), e14408. 10.1111/1751-7915.14408 38226780 PMC10832538

[B124] Tran T. T. Panesso D. Gao H. Roh J. H. Munita J. M. Reyes J. (2013). Whole-genome analysis of a daptomycin-susceptible Enterococcus faecium strain and its daptomycin-resistant variant arising during therapy. Antimicrob. Agents Chemother. 57 (1), 261–268. 10.1128/aac.01454-12 23114757 PMC3535923

[B125] Trofeit L. Sattler E. Künz J. Hilbert F. (2023). Salmonella prophages, their propagation, host specificity and antimicrobial resistance gene transduction. Antibiotics 12 (3), 595. 10.3390/antibiotics12030595 36978463 PMC10045043

[B126] Vandecraen J. Chandler M. Aertsen A. Van Houdt R. (2017). The impact of insertion sequences on bacterial genome plasticity and adaptability. Crit. Rev. Microbiol. 43 (6), 709–730. 10.1080/1040841X.2017.1303661 28407717

[B127] Varani A. He S. Siguier P. Ross K. Chandler M. (2021). The IS 6 family, a clinically important group of insertion sequences including IS 26. Mob. Dna 12, 11–18. 10.1186/s13100-021-00239-x 33757578 PMC7986276

[B128] Von Wintersdorff C. J. Penders J. Van Niekerk J. M. Mills N. D. Majumder S. Van Alphen L. B. (2016). Dissemination of antimicrobial resistance in microbial ecosystems through horizontal gene transfer. Front. Microbiol. 7, 173. 10.3389/fmicb.2016.00173 26925045 PMC4759269

[B129] Vrancianu C. O. Popa L. I. Bleotu C. Chifiriuc M. C. (2020). Targeting plasmids to limit acquisition and transmission of antimicrobial resistance. Front. Microbiol. 11, 761. 10.3389/fmicb.2020.00761 32435238 PMC7219019

[B130] Vranckx K. De Bruyne K. Pot B. (2017). “Analysis of MALDI‐TOF MS spectra using the BioNumerics software,” in MALDI‐TOF and tandem MS for clinical microbiology. Editors Shah H. N. Gharbia S. E. (Wiley), 539–562.

[B131] Wachino J. I. Jin W. Kimura K. Arakawa Y. (2019). Intercellular transfer of chromosomal antimicrobial resistance genes between Acinetobacter baumannii strains mediated by prophages. Antimicrob. Agents Chemother. 63 (8), e00334. 10.1128/aac.00334-19 31138576 PMC6658751

[B132] Waldor M. K. Mekalanos J. J. (1994). ToxR regulates virulence gene expression in non-O1 strains of *Vibrio cholerae* that cause epidemic cholera. Infect. Immun. 62 (1), 72–78. 10.1128/iai.62.1.72-78.1994 7903285 PMC186069

[B133] Wang L. Zhu M. Yan C. Zhang Y. He X. Wu L. (2023). Class 1 integrons and multiple mobile genetic elements in clinical isolates of the *Klebsiella pneumoniae* complex from a tertiary hospital in eastern China. Front. Microbiol. 14, 985102. 10.3389/fmicb.2023.985102 36950157 PMC10026359

[B134] Wang M. Liu P. Zhou Q. Tao W. Sun Y. Zeng Z. (2018). Estimating the contribution of bacteriophage to the dissemination of antibiotic resistance genes in pig feces. Environ. Pollut. 238, 291–298. 10.1016/j.envpol.2018.03.024 29573711

[B135] Wang Y. Lu J. Engelstädter J. Zhang S. Ding P. Mao L. (2020). Non-antibiotic pharmaceuticals enhance the transmission of exogenous antibiotic resistance genes through bacterial transformation. ISME J 14 (8), 2179–2196. 10.1038/s41396-020-0679-2 32424247 PMC7367833

[B136] Wang Y. Lu J. Zhang S. Li J. Mao L. Yuan Z. (2021). Non-antibiotic pharmaceuticals promote the transmission of multidrug resistance plasmids through intra-and intergenera conjugation. ISME J 15 (9), 2493–2508. 10.1038/s41396-021-00945-7 33692486 PMC8397710

[B137] Whitfield C. Wear S. S. Sande C. (2020). Assembly of bacterial capsular polysaccharides and exopolysaccharides. Annu. Rev. Microbiol. 74 (1), 521–543. 10.1146/annurev-micro-011420-075607 32680453

[B138] Willemsen A. Reid S. Assefa Y. (2022). A review of national action plans on antimicrobial resistance: strengths and weaknesses. Antimicrob. Resist. Infect. Control 11 (1), 90. 10.1186/s13756-022-01130-x 35739564 PMC9229779

[B139] Yang B. Wang Z. Jia Y. Fang D. Li R. Liu Y. (2022). Paclitaxel and its derivative facilitate the transmission of plasmid-mediated antibiotic resistance genes through conjugative transfer. Sci. Total Environ. 810, 152245. 10.1016/j.scitotenv.2021.152245 34896514

[B140] Yang X. Wai-Chi Chan E. Zhang R. Chen S. (2019). A conjugative plasmid that augments virulence in *Klebsiella pneumoniae* . Nat. Microbiol 4 (12), 2039–2043. 10.1038/s41564-019-0566-7 31570866

[B141] Yu G. Li Y. Liu X. Zhao X. Li Y. (2013). Role of integrons in antimicrobial resistance: a review. Afr J Microbiol Res 7 (15), 1301–1310. 10.5897/AJMR11.1568

[B142] Zaremba M. Dakineviciene D. Golovinas E. Zagorskaitė E. Stankunas E. Lopatina A. (2022). Short prokaryotic Argonautes provide defence against incoming mobile genetic elements through NAD+ depletion. Nat. Microbiol 7 (11), 1857–1869. 10.1038/s41564-022-01239-0 36192537

[B143] Zhang X. B. Oualline G. Shaw J. Yu Y. W. (2024). skandiver: a divergence-based analysis tool for identifying intercellular mobile genetic elements. Bioinformatics 40 (Suppl_2), ii155–ii164. 10.1093/bioinformatics/btae398 39230688 PMC11373320

[B144] Zheng Q. Daskalov A. (2023). Microbial gasdermins: more than a billion years of pyroptotic-like cell death. Semin. Immunol. 69, 101813. 10.1016/j.smim.2023.101813 37480832

[B145] Zomorodi A. R. Motamedifar M. Rahmanian K. Shakeri M. Hajikhani B. Heidari H. (2024). Investigation of integron classes 1, 2, and 3 among multi-drug-resistant *Staphylococcus aureus* isolates in Iran: a multi-center study. BMC Infect. Dis. 24, 1430. 10.1186/s12879-024-10311-5 39696000 PMC11653917

